# Using experience to create evidence: a mixed methods process evaluation of the new free family planning policy in Burkina Faso

**DOI:** 10.1186/s12978-022-01375-0

**Published:** 2022-03-18

**Authors:** Lalique Browne, Sarah Cooper, Cheick Tiendrebeogo, Frank Bicaba, Alice Bila, Abel Bicaba, Thomas Druetz

**Affiliations:** 1 School of Public Health, University of Montreal, C.P 6128, Succursale Centre-Ville, Montreal, QC H3C 3J7 Canada; 2 Société d’Études et de Recherche en Santé Publique (SERSAP), Ouagadougou, Burkina Faso; 3 Sciences de la vie et de la Santé, Université Aix-Marseille, Marseille, France; 4 Centre de Recherche en Santé Publique (CReSP), Montreal, QC Canada; 5 Center for Applied Malaria Research and Evaluation, Department of Tropical Medicine, Tulane University, New Orleans, LA USA

**Keywords:** Family planning, Implementation, User fee removal, Process evaluation, Health policy, Gratuity, Burkina Faso, Reproductive health, Planification familiale, mise en œuvre, exemption du paiement direct, évaluation de processus, politique de santé, Burkina Faso, santé reproductive

## Abstract

**Background:**

In 2019, Burkina Faso was one of the first countries in Sub-Saharan Africa to introduce a free family planning (FP) policy. This process evaluation aims to identify obstacles and facilitators to its implementation, examine its coverage in the targeted population after six months, and investigate its influence on the perceived quality of FP services.

**Methods:**

This process evaluation was conducted from November 2019 through March 2020 in the two regions of Burkina Faso where the new policy was introduced as a pilot. Mixed methods were used with a convergent design. Semi-directed interviews were conducted with the Ministry of Health (n = 3), healthcare workers (n = 10), and women aged 15–49 years (n = 10). Surveys were also administered to the female members of 696 households randomly selected from four health districts (n = 901).

**Results:**

Implementation obstacles include insufficient communication, shortages of consumables and contraceptives, and delays in reimbursement from the government. The main facilitators were previous experience with free healthcare policies, good acceptability in the population, and support from local associations. Six months after its introduction, only 50% of the surveyed participants knew about the free FP policy. Higher education level, being sexually active or in a relationship, having recently seen a healthcare professional, and possession of a radio significantly increased the odds of knowing. Of the participants, 39% continued paying for FP services despite the new policy, mainly because of stock shortages forcing them to buy their contraceptive products elsewhere. Increased waiting time and shorter consultations were also reported.

**Conclusion:**

Six months after its introduction, the free FP policy still has gaps in its implementation, as women continue to spend money for FP services and have little knowledge of the policy, particularly in the Cascades region. While its use is reportedly increasing, addressing implementation issues could further improve women’s access to contraception.

**Supplementary Information:**

The online version contains supplementary material available at 10.1186/s12978-022-01375-0.

## Plain Language summary

Burkina Faso is one of the first countries in sub-Saharan Africa to remove user fees for family planning services. Introduced as a pilot in June 2019, this policy covers the main costs, including the contraceptives, for all women of reproductive age (15–49 years old). We conducted a study to find out how the implementation of this new policy was going. In particular, we wanted to know what might be limiting or facilitating the successful implementation of the policy in a rural community. Through interviews with health staff and women, we found that about half of the women did not even know that family planning was now free, even though it had been free for more than six months. In addition, there were problems in the supply chain, which meant that contraceptive methods may have become free, but they were no longer available. On the other hand, the new policy has been generally well received by the public: previous similar initiatives seem to have facilitated implementation, as have awareness campaigns conducted by non-governmental organizations. With this information, the new policy can be improved to further enhance women's access to contraceptive methods in rural Burkina Faso.

**Supplementary Information:**

The online version contains supplementary material available at 10.1186/s12978-022-01375-0.

## Résumé

**Contexte:**

En 2019, le Burkina Faso a été l'un des premiers pays d'Afrique subsaharienne à introduire une politique de planification familiale (PF) gratuite. Cette évaluation de processus vise à identifier les obstacles et les facilitateurs de sa mise en œuvre, à examiner sa couverture dans la population ciblée après six mois et à étudier son influence sur la qualité perçue des services de PF.

**Méthodes:**

Cette évaluation de processus a été menée de novembre 2019 à mars 2020 dans les deux régions du Burkina Faso où la nouvelle politique a été introduite à titre pilote (Cascades et Centre-Ouest). Des méthodes mixtes ont été utilisées avec un devis convergent. Des entretiens semi-dirigés ont été menés auprès du ministère de la Santé (n = 3), des agents de santé (n = 10) et des femmes âgées de 15 à 49 ans (n=10). Des enquêtes ont également été administrées aux femmes membres de 696 ménages sélectionnés de façon aléatoire dans quatre districts sanitaires (n = 901).

**Résultats:**

Plusieurs obstacles à la mise en œuvre ont été identifiés : une communication insuffisante, des pénuries de consommables et de contraceptifs, et des retards dans le remboursement par le gouvernement. Les principaux éléments facilitateurs étaient une expérience antérieure des politiques de gratuité pour les soins de santé, une bonne acceptabilité par la population et le soutien des associations locales. Six mois après son introduction, seuls 50% des participantes interrogées connaissaient la politique de PF gratuite. Un niveau d'éducation plus élevé, le fait d'être sexuellement active ou en couple, le fait d'avoir récemment consulté un professionnel de santé et la possession d'une radio augmentaient significativement la probabilité de connaître la politique. Parmi les participantes, 39% continuaient à devoir payer pour les services de PF malgré la nouvelle politique, principalement en raison de ruptures de stock les obligeant à acheter les contraceptifs ailleurs. Une augmentation du temps d'attente et des consultations plus courtes ont également été signalées.

**Conclusion:**

Six mois après son introduction, la politique d’exemption du paiement direct pour la PF présente encore des lacunes dans sa mise en œuvre, car les femmes continuent de dépenser de l'argent pour les services de PF et connaissent peu la politique, en particulier dans la région des Cascades. Bien que son utilisation soit apparemment en hausse, la résolution des problèmes de mise en œuvre pourrait améliorer davantage l'accès des femmes à la contraception.

## Résumé pour le grand public

Le Burkina Faso est l'un des premiers pays d'Afrique subsaharienne à abolir les frais de consultation pour les services de planification familiale. Introduite à titre pilote en juin 2019, cette politique couvre les principaux coûts, y compris les contraceptifs, pour toutes les femmes en âge de procréer (15–49 ans). Nous avons mené une étude pour savoir comment se passait la mise en œuvre de cette nouvelle politique. En particulier, nous voulions savoir ce qui pouvait limiter ou faciliter la mise en œuvre réussie de cette politique dans une communauté rurale. Grâce à des entretiens avec le personnel de santé et les femmes, nous avons constaté qu'environ la moitié des femmes ne savaient même pas que la planification familiale était désormais gratuite, alors qu'elle l'était depuis plus de six mois. En outre, il y avait des problèmes dans la chaîne d'approvisionnement, ce qui signifie que les méthodes contraceptives étaient peut-être devenues gratuites, mais qu'elles n'étaient plus disponibles. D'un autre côté, la nouvelle politique a été généralement bien accueillie par le public : des initiatives similaires antérieures semblent avoir facilité la mise en œuvre, tout comme les campagnes de sensibilisation menées par des organisations non gouvernementales. Grâce à ces informations, la nouvelle politique peut être améliorée afin de renforcer l'accès des femmes aux méthodes contraceptives dans les zones rurales du Burkina Faso.

## Background

In Sub-Saharan Africa (SSA), out-of-pocket expenses for healthcare create a barrier in accessing health services by the population [1]. Family planning (FP), which refers to all contraceptive methods used by individuals and couples to anticipate their desired number of children [2], is no exception; in most SSA countries, women must pay for contraception [3]. The financial barrier to FP contributes to reducing FP usage, resulting in a high number of unintended pregnancies. Unintended pregnancies can be the cause of unsafe abortions, pregnancy in young or elderly women, and/or pregnancies that are too many or too close together, contributing to high rates of maternal mortality [4].

According to the World Bank statistics, only 30.1% of women aged 15–49 used a contraceptive method in 2020 in Burkina Faso. Although partially subsidized by the state (approximately $1.8 billion CFA Franc in 2019 or 3.1 million USD), the cost of FP remains high, especially for the poorest households [5]. Notably, 14.3% of women in the poorest quintile used an FP method, compared to 42.5% of the wealthiest quintile [6]. To improve access to family planning, user fees for these services were abolished in two regions of Burkina Faso (Cascades and Centre-Ouest) as a pilot implemented by the Government. In this pilot area, direct payment was removed in all public health facilities for the main costs related to FP services and methods. Prior to this pilot, the approximate costs of the most popular FP methods (i.e., masculine condoms, pills, implants)–if obtained at a public health facility–ranged from 10 to 1500 CFA franc (~ 0.02–2 USD), on top of consultation fees [7]. Studies conducted in Burkina Faso and in other SSA countries have identified the cost of FP services as one of the main barriers to their usage [8–10]. Other barriers include fear of side effects, misconceptions, sociocultural norms, gender roles, pressure from family members, lack of information, and hidden costs (transportation, opportunity costs of visiting a health facility) [11–13]. The relative importance of these financial barriers is still unknown with regard to FP. On the one side, abolishing user fees for maternal healthcare services has significantly and rapidly increased their use, and has reduced health inequities [14–16]. On the other side, while removing user fees reduces the financial barrier, it does not completely eliminate it; indirect costs, non-medical expenses, and under-the-table fees remain [11–13, 17].

To our knowledge, no study has examined the process of implementing a free FP policy, nor its effects on access. This is a significant knowledge gap, given the implementation problems that other user fee exemption policies have encountered, notably drug shortages, delays in the distribution of consumables, a potential decline in the perceived quality of free services, a perception of increased workload among healthcare workers (HCW), and insufficient communication [14, 18–21]. These issues were sometimes so significant that some exemption policies had to be discontinued or suspended by health personnel to limit patient influx in their health facilities, in Burkina Faso and elsewhere [14, 22]. The FP user fee exemption policy could face not only these known challenges, but also new issues, due to the sensitive nature of FP. Since Burkina Faso is one of the first countries in SSA to implement such a policy, little evidence is available. It is important to document the implementation process and share Burkina Faso's experience with countries seeking to implement a similar approach.

A process evaluation was conducted independently by this research team in the pilot area to provide scientific evidence to governmental health authorities and inform the implementation process of a future national policy removing user fees for FP services for all of Burkina Faso. The research team had no role in the planning or the implementation of the new policy. With the intention of maximizing the policy's impact on access to FP services, the objectives here are to assess: (i) the presence of obstacles and facilitators during the implementation, (ii) its coverage and implementation level in the targeted population, and (iii) its influence on the perceived quality of FP services.

## Methods

### Description of the free FP policy

**Fig. 1:**
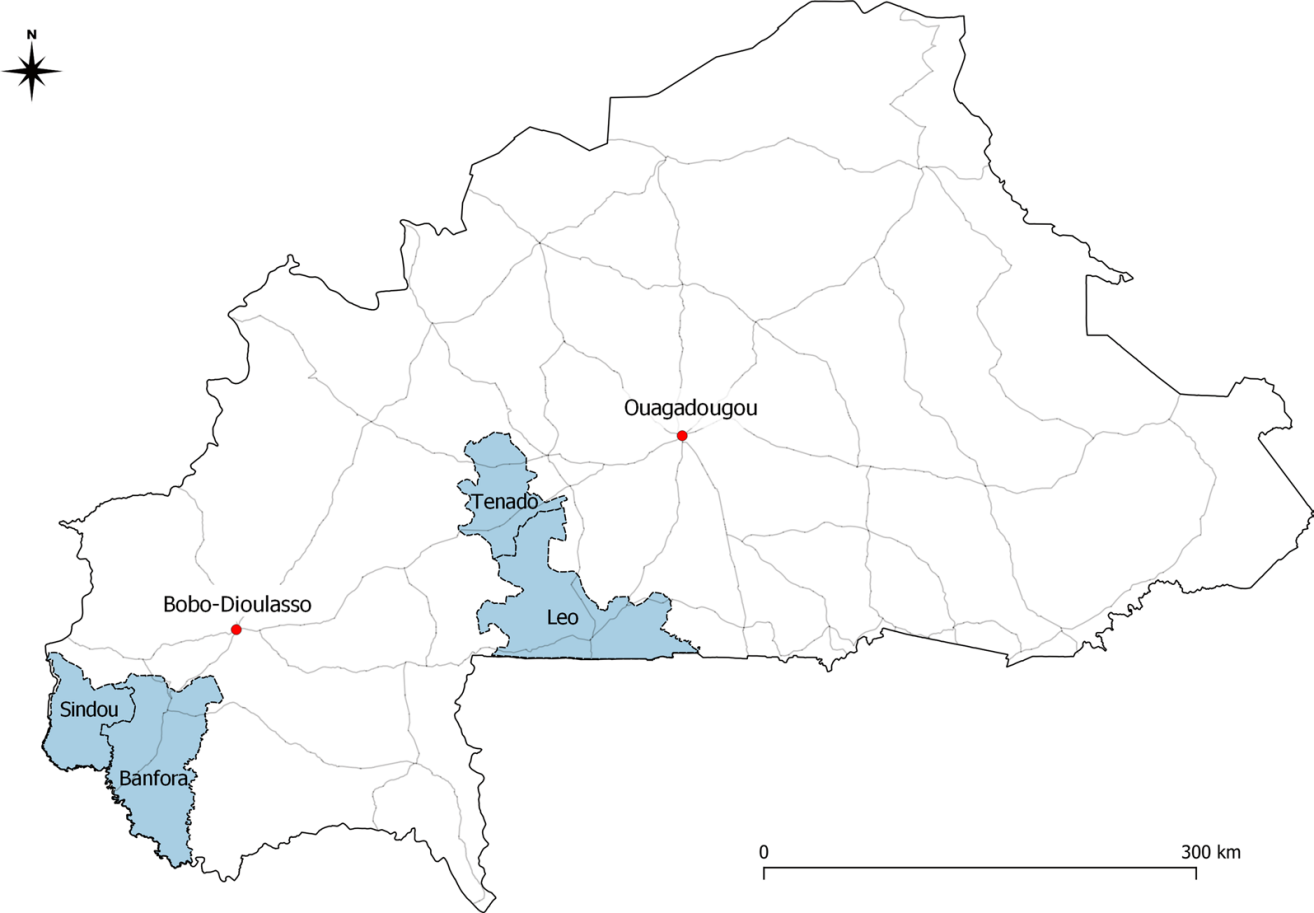
Map of study area. The four health districts are displayed in blue. Main roads are shown as grey lines

The free FP policy was introduced as a pilot in the Cascades and Centre-Ouest regions in June 2019 by the Government of Burkina Faso (see Fig. 1). These areas comprise a total population of ~ 2.5 million, mostly (> 80%) located in rural areas, and present a fertility rate of around six children per woman [21, 22]. The policy applied to all public health facilities and covered 100% of the cost of FP consultations and counseling, tests and examinations, and contraceptives themselves (injectables, implants, copper intrauterine devices, emergency contraceptive pills, condoms, surgical methods, and a range of natural methods). Management of side effects and transport to the reference health facility for medical evacuations were also covered, as were all FP-related medical procedures (e.g., installation and removal of implants) and consumables (gloves, syringes, swabs, disinfectants, etc.). The goal is for women who are sexually active to pay nothing for any aspect of FP.

The introduction of free FP followed a national policy implemented in July 2016 that removed user fees for maternal and child healthcare services [23]. Both interventions use the same third-party reimbursement mechanisms and follow similar administrative and reporting procedures. As such, the free FP policy was conceptualized as a functional scale-up of the national user fee removal policy [24]. The implementation process was therefore facilitated and consisted mainly of informing health personnel through official channels of the extension of free procedures to family planning-related services. Dissemination activities in the population were also planned through radio messages and awareness campaigns in the communities by healthcare providers.

### Study design

**Fig. 2:**
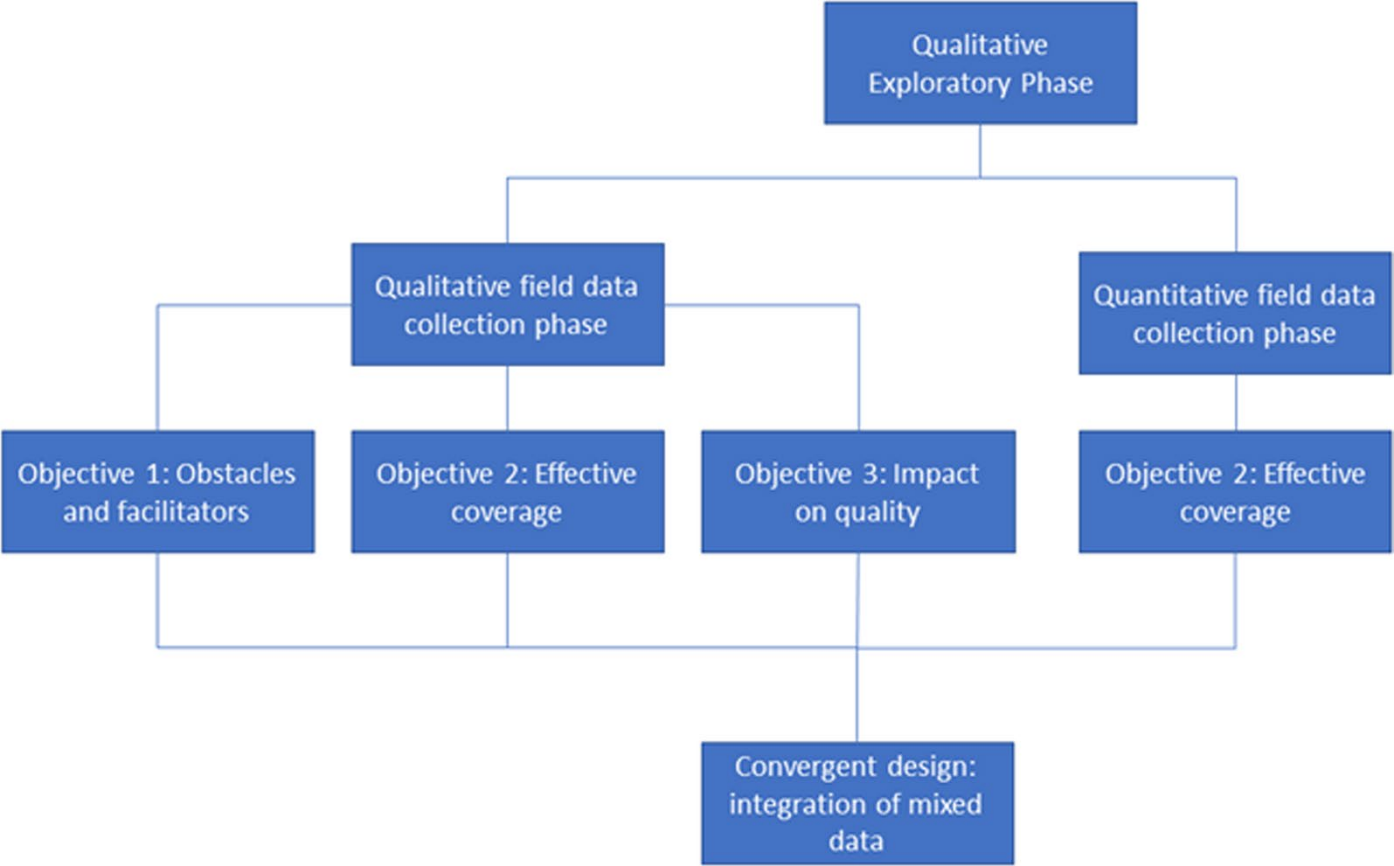
Study design and collection phases

This study was conducted between November 2019 and March 2020 in two separate phases (see Fig. 2). This study was conducted with the use of a mixed method design as per Creswell and Clark [25]. A mixed methods design was chosen in order to benefit from knowledge that comes from both qualitative and quantitative research as well as the integration of these two approaches [25]. First, a qualitative exploratory phase was undertaken to gain insight into the policy implementation process and refine research questions and instruments. Second, a qualitative and quantitative field data collection phase was conducted. Data was triangulated during the analysis using a convergent mixed method design, which was used to assess the coverage and implementation level of the free FP policy among the targeted population (Objective 2).

The other two objectives, to investigate the presence of obstacles or facilitators to implementation and to assess the policy's influence on the perceived quality of FP services, were pursued qualitatively. This was intentional, since it was necessary to gain an in-depth understanding of these topics and explore emerging themes—which is particularly suitable to qualitative research [26, 27]. To attain a variety of perspectives, qualitative investigations focused on three levels of policy implementation: (i) the central level, with the Ministry of Health (MoH); (ii) the peripheral level, with HCWs; and (iii) the community level, with direct beneficiaries of the policy. Moore's conceptual framework for process evaluations of complex interventions guided this implementation study (see Additional file 1: Appendix S1) [28]. The specific components of Moore’s conceptual framework studied in this evaluation are process (Objective 1), fidelity and reach (Objective 2), and outcomes (Objective 3).

The study took place in the context of rising insecurity in the country caused by terrorist attacks [29]. It was also conducted shortly after a nationwide strike had paralyzed non-essential activities in health facilities for several weeks.

### Qualitative exploratory phase

In October 2019, official documents (national planning and implementing strategy textbooks, information guides for health authorities, policy statements) were collected to gather as much information as possible before conducting interviews. In November 2019, semi-structured individual interviews (n = 3) were conducted in the capital Ouagadougou with program planners within the MoH involved in developing the free FP policy. The participants were conveniently selected with the assistance of a well-known knowledge broker for health matters in Burkina Faso. Interviews took place in MoH actors' offices. They were conducted in French by LB, were supervised by a local researcher trained in qualitative research (AB), and lasted 30–60 min. Field notes were taken during the interviews, which were audio-recorded. After three interviews, information collected was deemed sufficient to form a good understanding of how implementation of free FP had been planned. Data was interpreted based on the review of planning documents, field notes and audio recordings, and with feedback from the other research team members. The fact that many researchers on the team had been involved for several years with the MoH in studies on free healthcare policies enriched this exploratory phase.

### Quantitative field data collection phase

This study's quantitative component was embedded in another ongoing research project that aimed to evaluate the impacts of the national policy that removed health center user fees for pregnant women and children under five in 2016. Questions specific to the free FP policy were added to the original survey. The quantitative component was mostly designed to pursue Objective #2 of the present study: assessing the coverage and implementation level of the free FP policy among the targeted population. The coverage dimension was explored by assessing the beneficiaries' knowledge of the FP policy and its associated factors, while the implementation level was examined by considering the presence of residual costs related to FP.

#### Sampling

The sampling procedures were derived from those of the USAID Demographic and Health Surveys program. A two-stage cluster sampling was carried out in four out of 10 health districts: Leo and Tenado (Centre-Ouest) and Sindou and Banfora (Cascades). These districts were purposively selected based on two criteria: They contained health facilities located in rural areas, and they were secure (not having experienced any attacks since at least 2016). Using the enumeration areas as defined by the Demographic and Health Surveys Program in these four districts, 29 were randomly selected with a probability proportional to the size of their population. In the second stage, 24 households per unit were randomly selected with equal probability.

The target sample size of households was 696. Only households with ≥ 1 woman aged 15–49 were eligible. Ineligible households and households that could not be found were replaced by the nearest one.

#### Data collection

The survey took place in March 2020, after a five-day training for the interviewers. A questionnaire adapted from the standardized Demographic and Health Survey was administered to all consenting women aged 15–49 years from the selected households. It was administered in the local language by female interviewers with prior experience in community-based surveys. Although the standardized questionnaire already covered the participants' sociodemographic characteristics and use of FP services, some questions were added to record out of pocket payments for FP services covered by the policy and the participants' knowledge of the free PF policy.

Responses were collected electronically on tablets using Commcare software (Dimagi, Cambridge, USA). Questionnaire data was automatically uploaded to a secure server then extracted and cleaned using Stata 14.0 (StataCorp, College Station, TX).

#### Analysis

Descriptive analysis was performed on three key variables related to the implementation of the free FP policy. The coverage of this policy was first assessed by estimating the proportion of targeted women who know that FP was now officially free of charge at health facilities. Secondly, contraceptive use (and moment of procurement) was measured by categorizing women according to their current use of contraceptives (yes/no) and the moment they last procured them, i.e., before or after the policy had been implemented. Thirdly, the costs associated with the respondent’s last FP visit to the health facility were analyzed by the time of the visit (before or after the introduction of the free policy) and broken down by type of service.

All statistical analyses were conducted in open-source R statistical software V3.5.2. Maps were produced using the open-source software QGIS v3.8.1 Zanzibar. A multivariable logistic regression model with robust variance estimators was used to investigate factors related to knowledge of FP policy. The difference in the proportion of participants who paid for FP services before and after the introduction of the free policy was estimated by Chi-square tests of homogeneity of variance. The threshold for statistical significance was set at 0.05 (bilateral tests).

### Qualitative field data collection phase

#### Sampling

The qualitative component consisted of semi-directed individual interviews with health personnel and female community members. For convenience and logistical reasons, the qualitative research took place only in the district of Banfora, where five public health facilities were selected based on their location (accessible rural areas) and their type (health facilities without maternity services were excluded). In each health center, the head nurse and another member of the health staff (preferably a midwife) were individually interviewed (n = 10). With their assistance, households or groups of households with women of reproductive age were identified in the catchment area, and two women were selected in the community surrounding each health facility (n = 10). Selection was stratified by age, with one female community member < 20 and the other one > 20; these participants were from different households. To be selected, female community members had to be currently using FP.

#### Data collection

Data was collected in January 2020. For community members, the interviews took place outside of their home, in a secluded location that guaranteed the confidentiality of the respondents. For health personnel, interviews took place in a private room at the health facility. An interview guide specific to the type of the participant and with open-ended questions was used during the discussion (see Additional file 1). The interviews were conducted in French or in Djoula (depending on the participant's preference) by a single female researcher with extensive training in qualitative research.

Interviews with health personnel and women lasted 30–50 min and 15–30 min, respectively. They were recorded, transcribed verbatim and translated into French by an assistant. The field researcher listened to the original audio recordings and validated the transcripts. The research team members met once. Ten interviews were conducted with each type of participant and, with feedback from the researcher and her field notes, data saturation was considered to be reached.

### Analysis

A content analysis technique was carried out on the qualitative data. The transcripts were read several times for thorough understanding. The text was entirely segmented, and a mixed inductive and deductive coding was used: deductive because the coding grid was first established based on Moore's conceptual framework, and inductive because new codes were created for emerging themes [28]. The concepts from the framework which were used in the deductive coding grid were: process (obstacles and facilitators to the implementation), fidelity (payment for services or methods of FP), reach (knowledge of the intervention), and outcomes (perceived quality of care) [28]. Double coding was performed by two authors on a sample of the material to confirm the reliability of the final coding grid. Coding was conducted by LB and CB, as well as the thematic analysis. Peer debriefing was conducted with members of the research team, LB, CB and SC to derive themes from the codes. The interview results were triangulated with the data collected in the qualitative exploratory phase. The analysis was performed using QDA Miner software (QDA Miner 5.0).

### Convergence between qualitative and quantitative results

Integration of qualitative and quantitative data for the convergent design was carried out specifically for Objective 2 of our study: assessing coverage and implementation level of the free FP policy among the targeted population by investigating knowledge of the FP policy and residual costs related to FP. For this objective, quantitative and qualitative results were analyzed in parallel to study the same object before being integrated. A resulting comparison strategy was used by comparing the qualitative and quantitative components and identifying and interpreting divergences and convergences [25]. This interpretation was carried out to expand our understanding of conclusions on the free FP policy. For example, qualitative results were used to confirm our quantitative finding and explain specific quantitative results (expenditures on FP and knowledge level of the new policy).

### Ethical considerations

All participants provided informed written consent for both the qualitative and quantitative data collections. For the quantitative phase, consent was recorded on the tablet where the questionnaire was conducted. The questionnaire and the interviews were administered individually in a secluded area to preserve the confidentiality of participants. Participants aged 15–17 years old were considered mature minors and consented as adults. All the study procedures, including those for obtaining consent, were approved by the Comité d’éthique de la recherche en sciences de la santé at University of Montreal (Certificate #CERSES-20-146-D) and by the Comité d’éthique pour la Recherche en Santé in Burkina Faso (Deliberation #2018-6-075). The funder of the study had no role in study design, data collection, data analysis, data interpretation, or writing of the manuscript.

## Results

Twenty-three individuals participated in the qualitative component of the study: three MoH workers who were in charge of implementing free FP at the central level, 10 HCWs working in rural health centers, and 10 female community members (FCMs) from the surrounding area. HCWs had 1–15 years of work experience. Half of the FCMs were aged 20–45 years; they were housewives, farmers, or traders. The other half of the FCMs were all students all aged 19 years. Although eligible, no FCMs aged 15–18 years old was recruited.

A total of 901 women of reproductive age participated in the quantitative component and were administered the survey. Their main sociodemographic characteristics are presented in Table 1.

**Table 1:** Descriptive statistics of survey participants, by district

	District				P-value	Total (n = 901)
	Banfora (n = 511)	Sindou (n = 121)	Tenado (n = 70)	Leo (n = 199)		
Age (years)^a^					0.116	
15–25	197 (38.55)	40 (33.06)	25 (35.71)	75 (37.69)		337 (37.40)
26–35	179 (35.03)	41 (33.88)	16 (22.86)	57 (28.64)		293 (32.52)
36–55	134 (26.22)	39 (32.23)	29 (41.43)	60 (30.15)		262 (29.08)
Education					< 0.001	
No primary education	215 (42.07)	90 (74.38)	44 (62.86)	137 (68.84)		486 (53.94)
Primary education or higher	296 (57.92)	31 (25.62)	26 (37.14)	62 (31.16)		415 (46.06)
Has a paid job	242 (47.36)	50 (41.32)	19 (27.14)	95 (47.74)	0.010	406 (45.06)
Relationship status					< 0.001	
Single and not sexually active	53 (10.37)	10 (08.26)	9 (12.86)	14 (07.04)		86 (09.54)
Single and sexually active	83 (16.24)	11 (09.09)	4 (05.71)	9 (04.52)		107 (11.88)
Monogamous relationship	246 (48.14)	39(32.23)	28 (40.00)	110 (55.28)		423 (46.95)
Polygamous relationship	129 (25.24)	61 (50.41)	29 (41.43)	66 (33.16)		285 (31.63)
Previously gave birth	387 (84.68)	102 (91.89)	59 (96.72)	169 (91.35)	0.005	717 (88.08)
Currently using a method of FP	204 (43.31)	42 (37.84)	16 (25.40)	69 (38.76)	0.045	331 (40.22)
Previous FP use	213 (26.17)	24 (21.62)	8 (12.70)	48 (26.97)	0.090	203 (24.70)
Recent healthcare center visit	25 (04.90)	14 (11.67)	10 (14.29)	32 (16.33)	< 0.001	81 (09.04)
Recent home visit by health care professional	321 (62.82)	90 (74.38)	43 (61.43)	140 (70.35)	0.038	594 (65.93)
Wealth index^a^					< 0.001	
Q1 (poorest)	99 (24.57)	16 (16.67)	0	16 (11.19)		131 (18.99)
Q2	80 (19.85)	22 (22.92)	12 (25.00)	24 (16.78)		138 (20.00)
Q3	92 (22.83)	14 (14.58)	8 (16.67)	29 (20.28)		143 (20.72)
Q4	64 (15.88)	30 (31.25)	14 (29.17)	35 (24.48)		143 (20.72)
Q5 (wealthiest)	68 (16.87)	14 (14.58)	14 (29.17)	39 (27.27)		135 (19.56)
Possession of a radio	286 (69.93)	47 (48.96)	24 (50.00)	64 (44.44)	< 0.001	421 (60.40)
Size of household^b^					< 0.001	
1–5	175 (34.25)	22 (18.18)	17 (24.29)	41 (20.60)		255 (28.30)
6–10	248 (48.53)	59 (48.76)	31 (44.29)	103 (51.76)		441 (48.95)
> 10	87 (17.03)	40 (33.06)	22 (31.43)	55 (27.64)		204 (22.64)
Type of setting					< 0.001	
Urban	369 (72.21)	34 (28.10)	0	67 (33.67)		470 (52.16)
Rural, far from a health center (> 5 km)	43 (08.41)	60 (49.59)	0	83 (41.71)		186 (20.64)
Rural, close to a health center (< 5 km)	99 (19.37)	27 (22.31)	70 (100.00)	49 (24.62)		245 (27.19)

FP family planning; km kilometer; ^a^Data is missing for 9 participants; ^b^Data is missing for 1 participant

### Implementation barriers

Different sorts of barriers were mentioned during the interviews, depending on the participants. First, the MoH actors perceived important structural and transitory obstacles to the implementation of the new policy. Some of these were anticipated, such as opposition from Catholic leaders and religious associations. They also saw the risk of free family planning being misinterpreted as encouraging abortion, which would have met with resistance from the public. Others were contextual, like the climate of insecurity in the country that resulted in depreciated access of the population to the health facilities where FP services are offered and, vice versa, depreciated access of the health personnel to the communities. Moreover, the HCWs' strike in 2019 further limited the number of community-based activities performed by health personnel and left little space for communication around FP. These issues justified adjustments to policy planning, notably by reducing the intensity of the communication strategy. The MoH actors acknowledged that, in this particular context, they decided to keep a low profile to avoid protests. MoH actors felt that there was little official communication from the central level to the population and they did not expect the policy to produce a significant effect on FP usage.

*"People often mix up FP and abortion* […]*. It’s this confusion that often creates problems for us. Because as soon as we adopted free policy in December* […]*, we were called by the Catholic Church. I had to go to the cathedral to explain the issue related to free FP and give clarifications"* (MoH#1),

*''See, that's because the communication did not follow. Even you at the central level, you did not hear a lot. Because logically we should have communicated on this on all the radios, we should have talked''* (MoH#2).

*''Because people need to have the information that it's free. But the communication was more busy dealing with the strike. So I'm afraid that the results will be mitigated, that we won't really have a difference between the period before the free FP policy and the period during the free FP policy''* (MoH#2).

The health personnel pointed to stock shortages as the most important implementation obstacle. These shortages concerned mainly syringes, but also gloves, compresses, medical tape, and tweezers and scalpels to remove implants. Women confirmed that they experienced situations in which they had to pay to procure some consumables that were out of stock in the health facility.

*'' It was the syringe shortage.* […] *If someone comes for an implant insertion method, there are no syringes. So they have to go and buy it somewhere else''* (HCW#3).

*'' Sometimes there are no more gloves, you have to go and buy some''* (FCM#5, 45 years old)*.*

These shortages were reportedly triggered by the rapid increase in demand for FP services and contraceptives after the introduction of the policy. A few women also mentioned that they had experienced shortages in their FP method, although most providers reported these were less frequent than shortage in consumables. Another contributory factor was the slow reimbursement mechanism. Indeed, health facilities must absorb the costs of the offered FP services and then request to be reimbursed by the government; however, they sometimes experience a delay of several months before receiving reimbursement, putting them under financial pressure and contributing to stock shortages.

*"It is difficult to be reimbursed, which means that it is not easy to get money to buy supplies. Because if we don't reimburse you, we can't buy supplies"* (HCW#1).

These shortages are perceived as detrimental. Not only do they reduce access to free contraceptives, they also discourage beneficiaries to consistently use them. Local tensions and women's distrust towards the health personnel can also result from these situations.

*"For FP, you'll be out at least three or four days before you go place an order. These three days are not small. I take a Friday like that, because it's market day on Friday, a lot of women will come, if you happen to be out of the product, you know? So the interval of three, five days like that, before going to make the next order, especially in terms of FP, in any case it's felt. And then it discourages"* (HCW#2).

### Implementation facilitators

Several elements were favorable to the implementation of the new policy. Most importantly, once a year since 2012, there has been an initiative called National FP Week in Burkina Faso. During these specific weeks, all FP services were already offered free of charge. This initiative was described at both the central and peripheral levels as an opportunity for the population and health personnel to be familiarized with the future free FP policy.

Another key facilitator stems from the functional scaling-up strategy. Indeed, prior to the free FP initiative, user fees had already been removed in all public health facilities for pregnant women and children under five. The implementation of this new policy was facilitated since, from an institutional standpoint, new services were simply added to the basket of free services.

Contrary to the hypothesis formulated by MoH program planners at the central level, according to which the population's resistance to FP would have acted as an obstacle, FCM and HCW participants favored the free FP policy. FCM stated that FP allows them to rest by increasing birth spacing, and that the new policy is particularly helpful for women with limited financial means. HCWs agree, and confirm that they support the policy because it helps women in need and has positive health benefits.

Some participants also mentioned that FP is no longer taboo, and that community members, including men, are increasingly supportive.

*'' I think the men understood. They are starting to give the green light to the ladies to come and put on FP''* (HCW#3).

*'' I see that it helps women, we who are also in school, it helps us to continue our studies, our professional activities''* (FCM#2, 19 years old).

According to HCWs, the lower-than-anticipated resistance in the population can partly be attributed to the awareness-raising activities of several non-governmental organizations (NGOs) in the district, such as *Pathfinder*, *Marie Stopes International* and *Ma Copine*. These NGOs also reportedly facilitated the implementation of the new policy by relieving health personnel of some outreach community activities on FP.

### Knowledge of free FP policy

Overall, half (50.4%) of the survey participants knew about the new policy. The odds of knowing the existence of the new policy differed significantly between districts, even after adjusting for a set of potential confounding variables at the individual and household levels (Table 2). Compared to the most populated district (Banfora), the adjusted OR was lower in Sindou (aOR = 0.41, 95% CI [0.23–0.71]), and higher in Leo (aOR = 2.04, 95% CI [1.36–3.04]) and Tenado (aOR = 3.38, 95% CI [1.75–6.50]). The other spatial variables (i.e., urban vs. rural setting and the distance between the household and the nearest health center) were not significantly associated with knowledge of the new policy.

**Table 2:** Factors associated with knowledge of the new free family planning policy

	Knowledge of the new free FP policy			
	OR	95% CI	aOR	95% CI
Individual level				
Age (years)				
16–25 (ref)				
26–35	1.28	[0.93–1.75]	1.17	[0.77–1.76]
36–55	1.00	[0.72–1.38]	0.94	[0.61–1.45]
Received primary education	1.13	[0.87–1.47]	1.47*	[1.04–2.15]
Relationship status				
Single and not sexually active (ref)				
Single and sexually active	2.73	[1.50–5.06]	2.75**	[1.33–5.68]
Monogamous relationship	3.45	[2.10–5.84]	3.08**	[1.55–5.13]
Polygamous relationship	2.26	[1.35–3.88]	2.47*	[1.19–5.11]
Previous FP use	1.12	[0.82–1.54]	0.93	[0.65–1.33]
Recent health care center visit	1.97	[1.23–3.22]	1.81*	[1.07–3.08]
Recent home visit by health care professional	1.65	[1.25–2.19]	1.60**	[1.15–2.24]
Household level				
District				
Banfora (ref)				
Sindou	0.39	[0.24–0.60]	0.41*	[0.23–0.71]
Tenado	2.98	[1.74–5.31]	3.38***	[1.75–6.50]
Leo	2.08	[1.48–2.93]	2.04***	[1.36–3.04]
Wealth index				
Q1 (poorest) (ref)				
Q2	0.99	[0.65–1.50]	0.90	[0.55–1.45]
Q3	1.59	[1.05–2.41]	1.47	[0.89–2.43]
Q4	1.24	[0.82–1.88]	1.24	[0.72–2.13]
Q5 (wealthiest)	1.50	[0.99–2.28]	1.47	[0.86–2.54]
Possession of a radio	1.24	[0.95–1.62]	1.47*	[1.05–2.06]

FP, family planning; CI, confidence interval; OR, odds ratio; * p < 0.05 ** p < 0.01 *** p < 0.001; ref reference

Interviews confirmed that the majority of female community members had not been informed of the newly free services offered. Moreover, among those who knew, women mentioned not having enough information about what free FP entailed, especially about what services were included and if side effects of FP were covered by the policy.

*I don't have enough information on that* [Free FP policy]*. I only know that the FP methods are free. The health worker didn't say anything more"* (FCM#1, 20 years old)

"*I want more information about free FP or if for example I use a method and something happens to me as a result, how does it work?"* (FCM#5, 45 years old)

Apart from location, some sociodemographic characteristics were also associated with increased odds of knowing about the new policy (Table 2). In particular, the model suggests an increased odds ratio of knowing about the new policy if women are in a relationship or sexually active, if they received primary education, if they saw a healthcare worker in the last 12 months, and if their household owns a radio.

The importance of the radio for receiving information about free FP was also mentioned during interviews. Many participants reported having heard about the free FP policy through the radio.

" *[...] it is the local radio here that really helps us, that is really listened to by the population, that helps us in the messages it broadcasts.* '' (HCW#3)

"I heard about this on the radio". (TCM#2, 19 years old)

Some women also reported having received the information through their health center, by health agents. Transmission of information throughout the community by word of mouth or by village animators was also commonly mentioned.

“I went to the health center and the health agents informed us” (FCM#2, 38 years old).

“The people of my community informed me about it” (TCM#1, 19 years old).

“I think that the information also circulated through word of mouth” (HCW#5).

Finally, after adjusting for education and marital status, the model showed no association between knowledge of the new policy and the age of the participants. However, the interviews with teenagers revealed that they were particularly uninformed: Only one teenager out of the five interviewed was informed, at her school, of the free FP policy.

“They gave us the information through our school. It’s a doctor, even, who gave the information” (TCM#3, 19 years old).

### Cost of FP visits

About 66% (127/191) of the survey participants who obtained their most recent contraceptive method before the introduction of free FP had to pay for it. This proportion was reduced to 39% (45/115) for women who paid for their most recent contraceptive method after the introduction of the new policy (change: −27%, 95% CI [−15.5 to −39.2]). The reduction was three times larger in the Cascades region than in the Centre-Ouest region (Table 3).

**Table 3:** Proportion of women using contraception who had to pay for it, after vs. before the introduction of the policy, by region

	Before	After	Risk difference	95% CI	p- value
Cascades (Banfora & Sindou)	0.75	0.4	−0.35	[−0.452 to −0.237]	< 0.001
Center West (Tenado & Leo)	0.455	0.333	−0.121	[−0.228 to −0.75]	0.037

Proportions are displayed by region, rather than by districts, because of the small number of women using contraception in some districts

FP, family planning; CI, confidence interval

The MoH actors and HCWs stated during the interviews that all FP methods and services were now officially offered free of charge in their health facilities. On the other hand, similar to what was observed in the survey, interviewed women mentioned that they still had to pay for FP, even if it is officially free.

"*It's in effect, it's really free. When women come, they simply choose their method*" (HCW#3).

"*I heard about it at the radio. But when I went to the health center for my FP, it was not free anyways..*." (TCM#2, 19 years old, obtained her FP in December 2019).

Women gave further information about this situation and reported that, despite the policy, it was mostly the consumables that were not free, and sometimes the contraceptives themselves. This was acknowledged by HCWs, who explained that consumable or contraceptive shortages at the health facilities would force women to buy them elsewhere. Survey data support this finding; after the introduction of the policy, remaining costs related to FP were mostly for the contraceptive method itself, not for the consultation (Additional file 1: Appendix S3).

"*But often we are even forced to get supplies from the city's pharmacies. It's a bit complicated because* [...] *you can't give this to the clients for free. They have to pay the price of the dispensary*" (HCW#10).

*"When they* [healthcare workers] *say that they don't have these materials, they tell you to buy them elsewhere"* (FCM#2, 38 years old).

### Perceived quality of FP services

Overall, women are satisfied with the quality of FP services and mentioned having positive experiences with HCWs.

*"I have seen that in many health centers, health workers take good care of women. I am not going to lie. There is a gentleman even, he knows his job. He explains the use of contraceptives well"* (FCM#3, 40 years old)

*"The day I went, when my turn came, I went in alone. They gave me good advice. They explained everything to me"* (TCM#2, 19 years old)

However, some access issues reportedly attenuated the perceived quality of care, notably postponed appointments and long waiting periods. The HCWs acknowledged these issues, which, according to them, are attributable to the increased workload. They admitted being overwhelmed by the high demand for FP services, which forced them to increase the pace of consultations with women. This increased workload could have affected patient-provider relationships.

*"Others may come in the morning and the health workers have a lot to do, so they may ask to come back in the evening. Others also come without success and it is only the next day that they manage to get their contraceptive"* (FCM#5, 45 years old)

*"I waited a long time. I went in the morning around 6:00. When I came back, it was 1:00 p.m."* (FCM#2, 19 years old)

*"I can't lie to you, we don't have time to spend almost [an hour] with one patient, oh no, it's a little difficult. But to say that we're going to take time with a patient like we used to, it's going to be a little complicated"* (HCW#5)

*"Free FP came and the utilization rate of implant use tripled* [...]. *Women adhering to FP doubled during free FP* [...]. *Your mood will also change, and your reception will not be the same. The communication with the patients is not the same. So, it also has a negative influence"* (HCW#2)

In some facilities, HCWs also adapted their clinical practices by organizing group sessions rather than individual consultations. For example, women are sometimes gathered for FP counseling.

*"Yes, yes, if there are a lot of them like that* [several women to consult]*, we do group counseling at the moment"* (HCW#8)

## Discussion

To our knowledge, this study is the first to explore the process of implementing a free FP policy in SSA, to assess knowledge of the policy among the targeted rural population after six months, and to investigate its influence on the perceived quality of FP services. Although the implementation of free healthcare policies has already been examined in different settings, this study identified new factors that were at play and seem to be more specific to the removal of user fees for FP services.

Our study is consistent with the prediction that there would be increased in demand for FP services, and better access to them, after removing user fees. Similar to what was observed in many settings where free healthcare policies were introduced [7, 8, 14, 17], this immediate increase generated implementation barriers: drug shortages, delays in distribution of consumables, and perception of heavier workload in health workers. These barriers meant that women still had to pay (partially) for FP services even after the introduction of the new policy, although the likelihood was significantly reduced. Similar issues were observed after removing user fees for pregnant women and children; evidence gathered elsewhere suggests that these issues are transient and tend to gradually decrease under routine conditions of implementation [12, 13, 17]. On the road to routinization, the problem of sustainable funding should not be ignored; however, this policy is considered a national priority and is directly attributable to the Burkinabe regular budget [23].

While removing user fees is generally well received by users, policy planners expected some resistance from religious associations and the general population in the case of FP services, notably because they are sometimes confused with abortion. Our findings do not support this prediction and show that both women and HCWs favored the new policy. Participants reported that there was some opposition to FP in the general population (husbands, religious leaders, etc.) but no resistance to the new policy per se. Three key factors facilitated the implementation of the new policy, notably by improving its acceptance: the previous experience of National Free FP Week, the assistance of women’s groups and NGOs, and the fact that most of the administrative procedures were already in place (since it was a functional scale-up).

Although the policy appears to be widely accepted, caution is required before reaching any conclusions. Indeed, the policy has facilitated access to FP services, but previous literature suggests that user fee removal policies alone does not necessarily suggest increased women’s decision-making autonomy regarding FP [30]. Another study conducted in Burkina Faso after the removal of user fees for FP services reported increased marital tension [31]. In some instances, healthcare providers had to adapt their practices to guarantee women’s confidentiality and safety [31].

Our analysis indicates that knowledge about the new policy was poor among women of reproductive age (~50%), even 6 months after its introduction. After more than 10 years of research experience on free healthcare policies in Burkina Faso, this is the first time that we have observed this. Information about policies that introduce or remove user fees usually disseminates quickly, although misinterpretations and imperfect knowledge of the policy details are common [32–34]. This is likely due to health authorities’ decision not to widely disseminate the information that FP had become free, for fear of community resistance. The qualitative and quantitative analyses identified several factors that were positively associated with the knowledge of the new policy: possession of a radio, recent visit to a health facility or home visit by a healthcare professional, education level, and being married or sexually active. Even if the information was not officially broadcast on the radio, it was nevertheless circulated on community radio stations, which has proven to be an effective dissemination strategy in West African countries [35, 36]. Several complementary strategies could be used not only to increase the number of people who know about the policy, but also to engage with communities about FP. These potential strategies include community talks, meetings with village leaders and community health workers, use of “town criers,” and mobile text/audio messaging, among others [37], and should be adapted to the targeted population and its living environment [38, 39].

The women who were most informed about the policy tended to have a higher education level and to have more frequent contact with the health center. Results also suggest, although it is not statistically significant, that there is a gradient with socioeconomic status. This means that the benefits of the free healthcare policy in rural communities will arguably first go to more privileged women before reaching those who are less well off, which would increase health inequities [40, 41]. Similar manifestations of the reverse equity hypothesis have been reported after user fee removal for caesarean deliveries in Benin and Mali [42]. This is particularly problematic in the case of FP, since the unmet needs for these services are known to be higher among less affluent women [43, 44].

In the same vein, our results indicate that knowledge about the new policy was lower among adolescents and women who were not yet sexually active. For decades, interventions regarding FP in SSA have mostly focused on married, adult women. Numerous calls to depart from such a model have led to the adoption of a human rights-based framework to women’s health [45, 46]. In theory, the removal of user fees for FP services is aligned with such a framework, since it increases financial access using a population-based approach. In practice, adolescents and unmarried women risk being discriminated against in their right to access contraception, with many negative repercussions for their health [47]. Addressing this issue is of critical importance to promote women’s rights to reproductive health in an equitable manner, which is an obligation of governments.

Despite using a rich mixed methods approach to determine and understand the implementation level of the free FP policy in Burkina Faso, this study has some limitations. While the quantitative data was collected in four districts, the qualitative data was only collected in one. This made the integration of quantitative and qualitative data impossible for data disaggregated by district. Our results and interpretations may not be representative of the implementation processes in other areas where the pilot was introduced. It was not possible to interview young women (15–18 years old) who were using contraception, which precludes an in-depth understanding of their perceptions about the policy. Also, even if they were not individually referred to us, female community members were recruited for an interview with the assistance of health personnel, which carries a risk of selection bias. For logistical reasons, this study focused on perceived quality of FP services rather than using an objective quality assessment method. Combining the surveys and interviews with direct observation could have produced a better representation of quality. A Hawthorne effect cannot be ruled out, and it is possible that a social desirability bias affected the participants’ answers on sensitive topics [48]. To lower this risk, individual interviews were performed by female fieldworkers highly trained in qualitative research methods. A recall bias is also plausible, notably for questions about women’s practices before the implementation of the policy. A potential way to limit this bias would have been to have baseline interviews prior to the implementation of the policy. However, the study took place as rapidly as possible (6 months) after the introduction to reduce this potential bias.

### Recommendations

Since 2016, the MoH in Burkina Faso has successively removed user fees for children under five years old, for maternal and reproductive healthcare, and now for FP services, in all public healthcare facilities. The positive effects of such initiatives in improving maternal and child health and reducing health inequalities should be highlighted. Based on the present study’s findings, it seems particularly important to increase awareness or knowledge of the FP policy among vulnerable groups such as adolescents, unmarried women, and women of lower socioeconomic status. These strategies should go beyond disseminating messages on the radio, either officially or informally. It also appears that misconceptions about FP persist, and that is still sometimes confused with abortion. But these misconceptions should not prevent progress in promoting women's right to sexual and reproductive health. The successful implementation of this policy in Burkina Faso shows that, while combating these misconceptions, it is possible to promote women's right to access family planning services. However, greater access does not automatically mean greater decision-making autonomy, and complementary measures to promote women's empowerment remain essential.

## Conclusion

Six months after its introduction as a pilot in two regions, the free FP policy in Burkina Faso has been facing implementation barriers that likely reduce its effectiveness, notably stock shortages, delays in reimbursements, and insufficient communication. It is concerning that knowledge about the new policy is lower among adolescents, unmarried women, and women with lower socioeconomic status. Hopefully, these barriers will be transient and will not increase health inequities. The acceptability of the new policy was facilitated thanks to sensitization campaigns and previous experiences during the Annual Free FP Week. Findings suggest an increase in the number of women using FP services, in both rural and urban areas. While the new policy is aligned with the promotion of women’s right to reproductive health, further reflection is required to reconcile better access to FP services and improved empowerment for women.

## Supplementary Information

**Additional file 1: Appendix S1. **Conceptual framework: process evaluation of complex interventions. **Appendix S2. **Interview guides. **Appendix S3. **Type of costs associated with FP, before and after implementation of policy.

**Additional file 2. ** French version of the article.

## Data Availability

All anonymized data can be made available by contacting the corresponding author under reasonable request.

## Abbreviations

aOR: Adjusted odds ratio
CI: Confidence interval
FCM: Female community member
FP: Family Planning
HCWs: Healthcare workers
MoH: Ministry of Health
NGO: Non-governmental organizations
SSA: Sub-Saharan Africa
