# Supplementary material for: Using experience to create evidence: a mixed methods process evaluation of the new free family planning policy in Burkina Faso
Source: Reprod Health. 2022 Mar 18;19:67. doi: 10.1186/s12978-022-01375-0 (PMC8932047; doi:10.1186/s12978-022-01375-0)
Supplement: Supplementary file 1 — Additional file 1: Appendix S1. Conceptual framework: process evaluation of complex interventions. Appendix S2. Interview guides. Appendix S3. Type of costs associated with FP, before and after implementation of policy. [file 12978_2022_1375_MOESM1_ESM.docx]

**Appendices**

**Appendix S1 Conceptual framework: process evaluation of complex interventions**


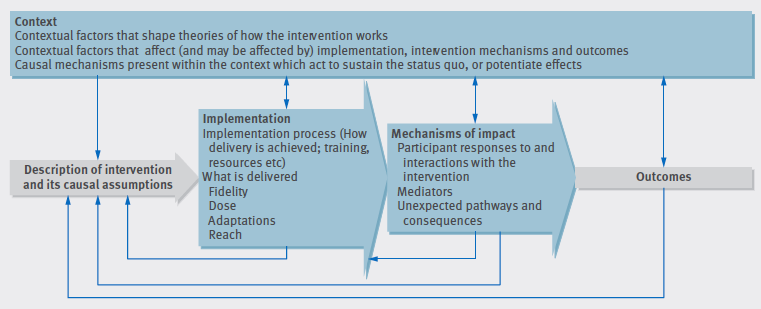


Source: Moore G F, Audrey S, Barker M, Bond L, Bonell C, Hardeman W et al. Process evaluation of complex interventions: Medical Research Council guidance, *BMJ* 2015; 350:h1258.

**Appendix S2 Interview guides**

Interview guide for healthcare workers

| **Objectifs** | **Questions** | **Sous-questions** |
| --- | --- | --- |
| Question introductive | 1) Comment se déroule la mise en œuvre de la politique de gratuité de la PF dans votre établissement de santé? |  |
| Documenter l’effectivité des mesures offertes par la politique | 2) Quelles méthodes de PF sont offertes gratuitement dans votre établissement? | **CSPS**   - Pilule - Injectables - DIU au cuivre - Implants - Préservatifs - Méthodes naturelles |
|  | 3) Quel panier de services de PF est offert dans votre établissement? | **CSPS**   - Les consultations et le counseling - La prise en charge clinique des effets secondaires et des complications de la PF - Examens complémentaires - Retrait ordinaire d’implants et de DIU - Carburant en cas d’évacuation |
| Documenter la présence de changements dans la qualité des services offerts depuis l’introduction de la politique | 4) Avez-vous observé des changements en termes de volume de consultation chez les femmes depuis l’introduction de la gratuité? Lesquels? | - Augmentation des files d’attente? - Augmentation de votre charge de travail? - Pas de changement? |
|  | 5) Si oui, est-ce que les changements dans le volume de consultation ont une influence sur les services de consultations et counseling offerts? | - Disponibilité des services de PF (files d’attente) - Temps passé en consultation avec le bénéficiaire - Adéquation des informations données au bénéficiaire |
|  | 6) Avez-vous observé des changements dans la disponibilité des contraceptifs et des consommables (gants, seringues, compresses) dans votre établissement depuis l’introduction de la gratuité de la PF? | - Avez-vous vécu des ruptures de stock depuis la gratuité? - Est-ce plus fréquent depuis la gratuité? - Pour quelle méthode? - Pour quel consommable? |
| Documenter la présence de facteurs contextuels (obstacles et facilitateurs) à l’implantation de la politique | 7) Que pensez-vous de cette politique de gratuité de la PF? | - Augmentation de votre charge de travail? - Efficacité / utilité? |
|  | 8) Qu’est-ce que les gens pensent de la politique de gratuité de la PF en général? | - Les femmes? - Les maris? - La communauté? |
|  | 9) Avez-vous rencontré des obstacles lors de l’implantation de la politique dans votre CSPS? Lesquels? | - Grève? - Résistance des prestataires? - Acceptabilité de la population? - Insuffisance de communication du niveau central? - Incompréhensions? |
|  | 10) Dans votre district, y a-t-il des éléments particuliers qui pourraient avoir influencé la mise en œuvre harmonieuse de la politique de gratuité de la PF? | - Socioculturel? - Organisationnel? - Économique? - Communicationnel? - Autre? |
|  | 11) Est-ce que certains éléments ont rendu l’implantation de la politique dans votre CSPS plus facile? | - Connaissance préalable du mécanisme de gratuité - Prestation de services de PF déjà en place - Mécanisme d’approvisionnement des consommables déjà en place - Semaines de la PF préalablement effectuées |
| Conclusion | 12) Quelles recommandations feriez-vous au gouvernement pour améliorer la mise œuvre de la politique? |  |

Interview guide for female community members (women aged 15-49)

| **Objectifs** | **Questions** | **Sous-questions** |
| --- | --- | --- |
| Questions introductives | 1) À quand remonte votre dernière consultation des services de santé pour la planification familiale? |  |
| Documenter le niveau de connaissance de la politique par les femmes ciblées par celle-ci | 2) Savez-vous que la PF devrait désormais être gratuite dans les établissements de santé de votre région? | Si oui, comment le savez-vous?   - Affiches - Spot radio - Émission télé - Journée d’information - SMS - Crieurs publics - Agents de santé - Bouche à oreille |
|  | 3) Que savez-vous de la politique de gratuité de la PF? | Quels services sont inclus dans celle-ci?   - Méthode de PF - Consultations et counseling? - Prise en charge des effets secondaires et complications? - Matériel   Savez-vous que tout devrait être gratuit dans les établissements publics de santé? |
|  | 4) Jugez-vous que votre niveau de connaissance de la politique de gratuité de la PF est suffisante? | - Aimeriez-vous en savoir plus? - Quelles informations vous manque-t-il? |
| Documenter La présence de changements dans la qualité des services de PF depuis l’instauration de la politique | 5) Depuis juillet 2019, avez-vous observé des changements dans les services de PF dans les centres de santé? | - Est-ce que les files d’attente ont allongé? - Quelles conséquences cela a-t-il sur vous? - Est-ce qu’on passe moins de temps avec vous durant les consultations ou counseling? - Est-ce qu’on vous transmet moins d’informations? - Y-a-t ’il eu un changement au niveau de votre satisfaction des consultations de PF? |
|  | 6) Depuis juillet 2019, vous est-il arrivé que votre méthode de PF ne soit pas disponible? | - Si oui, cela arrive t’-il plus souvent qu’avant juillet 2019? - Quelle méthode? - Pour combien de temps n’était-elle pas disponible? |
|  | 7) Depuis juillet 2019, est-il arrivé que le matériel nécessaire à l’implantation de votre méthode contraceptive ne soit pas disponible (seringues, gants, compresses)? | - Si oui, cela arrive t’-il plus souvent qu’avant juillet 2019? - Quel matériel? - Pour combien de temps n’était-il pas disponible? - Avez-vous du déboursé pour obtenir ce matériel? |
| Documenter l’acceptabilité de la population envers la politique | 8) Quel est votre opinion de la politique de gratuité de la PF? | - Points positifs - Points négatifs |
|  | 9) Qu’est-ce que votre mari / conjoint pense de la politique? Qu’est-ce que votre communauté ou les autres femmes en pensent? | - Est-ce que certains s’opposent à la politique? - Comment s’y opposent-ils? |
| Conclusion | 10) Quelles est votre satisfaction générale de la politique? | - Qualité des services offerts - Disponibilité des services - Accessibilité des services - Considérez-vous que certains aspects pourraient être améliorés? Si oui, quoi? |

**Appendix S3 Type of costs associated with FP, before and after implementation of policy**

|  | Last date of obtaining contraception | |  |
| --- | --- | --- | --- |
| What did you spend money on? ^¶^ | >6 months ago | <6 months ago | All |
| Fee for consultation | 40 (0.32) | 8 (0.18) | 48 (0.28) |
| Material and examinations | 35 (0.28) | 8 (0.18) | 43 (0.25) |
| Drugs and contraception | 77 (0.61) | 33 (0.73) | 110 (0.64) |
| Other | 0 | 1 (0.02) | 1 (0.01) |

¶ Several answers are possible
