# Supplementary material for: Using experience to create evidence: a mixed methods process evaluation of the new free family planning policy in Burkina Faso
Source: Reprod Health. 2022 Mar 18;19:67. doi: 10.1186/s12978-022-01375-0 (PMC8932047; doi:10.1186/s12978-022-01375-0)
Supplement: Supplementary file 2 — Additional file 2. French version of the article. [file 12978_2022_1375_MOESM2_ESM.docx]

**Titre**

Une évaluation de processus de la nouvelle politique d’exemption du paiement direct pour la planification familiale au Burkina Faso

**Authors**

Lalique Browne^☯1^, Sarah Cooper^☯1^, Cheick Tiendrebeogo^1^, Frank Bicaba^2,3^, Alice Bila^2^, Abel Bicaba^2^, Thomas Druetz^1,4,5^*

^1^ École de Santé Publique, Université de Montréal, Montréal, QC, Canada

^2^ Société d’Études et de Recherche en Santé Publique (SERSAP), Ouagadougou,

Burkina Faso

^3^ Sciences de la vie et de la Santé, Université Aix-Marseille, Marseille, France

^4^ Centre de Recherche en Santé Publique (CReSP), Montréal, QC, Canada

^5^ Center for Applied Malaria Research and Evaluation, Département de Médecine Tropicale,

Université Tulane, La Nouvelle-Orléans, LA, USA

^☯^ Contribution égale

* Auteur de correspondance. Email : [thomas.druetz@umontreal.ca](mailto:thomas.druetz@umontreal.ca). Adresse : Université de Montréal, C.P 6128, succursale Centre-ville Montréal QC Canada H3C 3J7. ORCID: 0000-0002-9234-4286.

**Résumé**

Contexte. En 2019, le Burkina Faso a été l'un des premiers pays d'Afrique subsaharienne à introduire une politique de planification familiale (PF) gratuite. Cette évaluation de processus vise à identifier les obstacles et les facilitateurs de sa mise en œuvre, à examiner sa couverture dans la population ciblée après six mois et à étudier son influence sur la qualité perçue des services de PF.

Méthodes. Cette évaluation de processus a été menée de novembre 2019 à mars 2020 dans les deux régions du Burkina Faso où la nouvelle politique a été introduite à titre pilote (Cascades et Centre-Ouest). Des méthodes mixtes ont été utilisées avec un devis convergent. Des entretiens semi-dirigés ont été menés auprès du ministère de la Santé (n=3), des agents de santé (n=10) et des femmes âgées de 15 à 49 ans (n=10). Des enquêtes ont également été administrées aux femmes membres de 696 ménages sélectionnés de façon aléatoire dans quatre districts sanitaires (n=901).

Résultats. Plusieurs obstacles à la mise en œuvre ont été identifiés : une communication insuffisante, des pénuries de consommables et de contraceptifs, et des retards dans le remboursement par le gouvernement. Les principaux éléments facilitateurs étaient une expérience antérieure des politiques de gratuité pour les soins de santé, une bonne acceptabilité par la population et le soutien des associations locales. Six mois après son introduction, seuls 50% des participantes interrogées connaissaient la politique de PF gratuite. Un niveau d'éducation plus élevé, le fait d'être sexuellement active ou en couple, le fait d'avoir récemment consulté un professionnel de santé et la possession d'une radio augmentaient significativement la probabilité de connaître la politique. Parmi les participantes, 39% continuaient à devoir payer pour les services de PF malgré la nouvelle politique, principalement en raison de ruptures de stock les obligeant à acheter les contraceptifs ailleurs. Une augmentation du temps d'attente et des consultations plus courtes ont également été signalées.

Conclusion. Six mois après son introduction, la politique d’exemption du paiement direct pour la PF présente encore des lacunes dans sa mise en œuvre, car les femmes continuent de dépenser de l'argent pour les services de PF et connaissent peu la politique, en particulier dans la région des Cascades. Bien que son utilisation soit apparemment en hausse, la résolution des problèmes de mise en œuvre pourrait améliorer davantage l'accès des femmes à la contraception.

**Mots-clé**

Planification familiale, mise en œuvre, exemption du paiement direct, évaluation de processus, politique de santé, Burkina Faso, santé reproductive

**Résumé pour le grand public**

Le Burkina Faso est l'un des premiers pays d'Afrique subsaharienne à abolir les frais de consultation pour les services de planification familiale. Introduite à titre pilote en juin 2019, cette politique couvre les principaux coûts, y compris les contraceptifs, pour toutes les femmes en âge de procréer (15-49 ans). Nous avons mené une étude pour savoir comment se passait la mise en œuvre de cette nouvelle politique. En particulier, nous voulions savoir ce qui pouvait limiter ou faciliter la mise en œuvre réussie de cette politique dans une communauté rurale. Grâce à des entretiens avec le personnel de santé et les femmes, nous avons constaté qu'environ la moitié des femmes ne savaient même pas que la planification familiale était désormais gratuite, alors qu'elle l'était depuis plus de six mois. En outre, il y avait des problèmes dans la chaîne d'approvisionnement, ce qui signifie que les méthodes contraceptives étaient peut-être devenues gratuites, mais qu'elles n'étaient plus disponibles. D'un autre côté, la nouvelle politique a été généralement bien accueillie par le public : des initiatives similaires antérieures semblent avoir facilité la mise en œuvre, tout comme les campagnes de sensibilisation menées par des organisations non gouvernementales. Grâce à ces informations, la nouvelle politique peut être améliorée afin de renforcer l'accès des femmes aux méthodes contraceptives dans les zones rurales du Burkina Faso.

**Introduction**

En Afrique subsaharienne (ASS), les frais de santé déboursés directement par les patients constituent un obstacle à l'accès aux services de santé pour la population (1). La planification familiale (PF), qui désigne l'ensemble des méthodes contraceptives utilisées par les individus et les couples pour anticiper le nombre d'enfants qu'ils souhaitent (2), ne fait pas exception à la règle ; dans la plupart des pays d'ASS, les femmes doivent payer leur contraception (3). L'obstacle financier à la PF contribue à réduire son utilisation, ce qui entraîne un nombre élevé de grossesses non désirées. Les grossesses non planifiées peuvent être à l'origine d'avortements non sécurisés, de grossesses chez des femmes jeunes ou âgées, ou de grossesses trop nombreuses ou trop rapprochées, contribuant ainsi à des taux élevés de mortalité maternelle (4).

Selon les statistiques de la Banque mondiale, seulement 30,1% des femmes âgées de 15 à 49 ans utilisaient une méthode contraceptive en 2020 au Burkina Faso. Bien que partiellement subventionné par l'État (environ 1,8 milliard de francs CFA en 2019 ou 3,1 millions USD), le coût de la PF reste élevé, notamment pour les ménages les plus pauvres (5). Ainsi, 14,3% des femmes du quintile le plus pauvre utilisaient une méthode de PF, contre 42,5% du quintile le plus riche (6). Pour améliorer l'accès à la planification familiale, les frais d'utilisation de ces services ont été supprimés dans deux régions du Burkina Faso (Cascades et Centre-Ouest) dans le cadre d'un projet pilote mis en œuvre par le gouvernement. Dans cette région pilote, le paiement direct a été supprimé dans tous les établissements de santé publique pour les principaux coûts liés aux services et méthodes de PF. Avant ce projet pilote, les coûts approximatifs des méthodes de PF les plus populaires (c'est-à-dire les préservatifs masculins, les pilules, les implants) - si elles étaient obtenues dans un établissement de santé publique - allaient de 10 à 1500 francs CFA (~ 0,02-2 USD), en plus des frais de consultation (7). Des études menées au Burkina Faso et dans d'autres pays d'Afrique subsaharienne ont identifié le coût des services de PF comme l'un des principaux obstacles à leur utilisation (8-10). Parmi les autres obstacles figurent la peur des effets secondaires, les idées fausses ou rumeurs, les normes socioculturelles, les rapports de pouvoir entre les hommes et les femmes, la pression exercée par les membres de la famille, le manque d'information et les coûts résiduels cachés (transport, coûts d'opportunité de la visite d'un établissement de santé) (11-13). L'importance relative de ces obstacles financiers est encore inconnue en ce qui concerne la PF. D'une part, la suppression des frais d'utilisation des services de santé maternelle a permis d'augmenter rapidement et de manière significative leur utilisation et de réduire les inégalités en matière de santé (14-16). D'autre part, si la suppression des frais d'utilisation réduit l'obstacle financier, elle ne l'élimine pas complètement ; les coûts indirects, les dépenses non médicales et les frais officieux (tels que les pourboires exigés) demeurent (11-13, 17).

À notre connaissance, aucune étude n'a examiné le processus de mise en œuvre d'une politique de gratuité de la PF, ni ses effets sur l'accès. Il s'agit d'une lacune importante dans les connaissances, étant donné les problèmes de mise en œuvre que d'autres politiques d'exemption du paiement direct ont rencontrés, notamment des pénuries de médicaments, des retards dans la distribution des consommables, une baisse potentielle de la qualité perçue des services gratuits, une perception d'une charge de travail accrue parmi les travailleurs de la santé (TS) et une communication insuffisante (14, 18-21). Ces problèmes étaient parfois si importants que certaines politiques d'exemption ont dû être interrompues ou suspendues par le personnel de santé pour limiter l'afflux de patients dans leurs établissements de santé, au Burkina Faso comme ailleurs (14, 22). La politique d'exemption du paiement direct pour la PF pourrait faire face non seulement à ces défis connus, mais aussi à de nouveaux problèmes, en raison de la nature sensible de la PF. Le Burkina Faso étant l'un des premiers pays d'Afrique subsaharienne à mettre en œuvre une telle politique, peu de données sont disponibles. Il est important de documenter le processus de mise en œuvre et de partager l'expérience du Burkina Faso avec les pays qui cherchent à mettre en œuvre une approche similaire.

Une évaluation de processus a été menée indépendamment par cette équipe de recherche dans la zone pilote afin de fournir des preuves scientifiques aux autorités sanitaires gouvernementales et d'accompagner le processus de passage à l’échelle, en prévision d'une future politique nationale supprimant les frais d'utilisation des services de PF pour l'ensemble du Burkina Faso. L'équipe de recherche n'a joué aucun rôle dans la planification ou la mise en œuvre de la nouvelle politique. Dans l'intention de maximiser l'impact de la politique sur l'accès aux services de PF, les objectifs de cette étude sont d'évaluer : (i) la présence d'obstacles et de facilitateurs lors de la mise en œuvre (ii) sa couverture et son niveau de mise en œuvre dans la population ciblée, et (iii) son influence sur la qualité perçue des services de PF.

**Méthodes**

**La politique d’exemption du paiement direct pour la PF**

La politique d’exemption du paiement direct pour la PF a été introduite à titre pilote dans les régions des Cascades et du Centre-Ouest en juin 2019 par le gouvernement du Burkina Faso (voir Figure 1). Ces régions comprennent une population totale de ~2,5 millions d'habitants, majoritairement (>80%) située en zone rurale, et présentent un taux de fécondité d'environ six enfants par femme (21, 22). La politique s'appliquait à tous les établissements de santé publique et couvrait 100 % du coût des consultations et des conseils en matière de PF, des tests et des examens, ainsi que des contraceptifs eux-mêmes (injectables, implants, dispositifs intra-utérins en cuivre, pilules contraceptives d'urgence, préservatifs, méthodes chirurgicales et toute une série de méthodes naturelles). La prise en charge des effets secondaires et le transport vers la structure sanitaire de référence en cas d'évacuation médicale étaient également couverts, ainsi que tous les actes médicaux liés à la PF (par exemple, la pose et le retrait des implants) et les consommables (gants, seringues, écouvillons, désinfectants, etc.). L'objectif était que les femmes sexuellement actives ne paient rien pour aucun aspect de la PF.

L'introduction de la gratuité de la PF a fait suite à une politique nationale, mise en œuvre en juillet 2016, qui a supprimé le paiement direct pour tous les services de santé maternelle et infantile au Burkina Faso (23). Les deux interventions utilisent le même mécanisme de remboursement par tiers payant et suivent des procédures administratives et de déclaration similaires. À ce titre, la politique de PF gratuite a été conceptualisée comme une mise à l'échelle fonctionnelle de la politique nationale de suppression des frais d'utilisation (24). Le processus de mise en œuvre a donc été facilité et a consisté principalement à informer le personnel de santé par les canaux officiels de l'extension de la gratuité des procédures aux services liés à la PF. Des activités de diffusion dans la population étaient également prévues par le biais de messages radio et de campagnes de sensibilisation dans les communautés par les prestataires de soins.

**Conception de l'étude**

Cette étude a été menée entre novembre 2019 et mars 2020 en deux phases distinctes (voir figure 2). Un devis par méthode mixte selon Creswell & Clark (25) a été choisi afin de pouvoir bénéficier des connaissances issues de la recherche qualitative et quantitative, ainsi que de l'intégration de ces deux approches (25). Tout d'abord, une phase exploratoire qualitative a été entreprise pour mieux comprendre le processus de mise en œuvre de la politique et affiner les questions et les instruments de recherche. Ensuite, une phase de collecte de données qualitatives et quantitatives sur le terrain a été menée. Les données ont été triangulées au cours de l'analyse par la méthode de convergence, utilisée pour évaluer le niveau de mise en œuvre de la politique de gratuité de la PF parmi la population cible (objectif 2).

Les deux autres objectifs, à savoir étudier la présence d'obstacles ou de facilitateurs à la mise en œuvre et évaluer l'influence de la politique sur la qualité perçue des services de PF, ont été poursuivis de manière qualitative. C'était intentionnel, car il était nécessaire d'acquérir une compréhension approfondie de ces sujets et d'explorer les thèmes émergents - ce qui se prête particulièrement à la recherche qualitative (26, 27). Pour obtenir une variété de perspectives, les enquêtes qualitatives se sont concentrées sur trois niveaux de mise en œuvre des politiques : (i) le niveau central, avec le ministère de la Santé (MS) ; (ii) le niveau périphérique, avec les travailleurs de santé ; et (iii) le niveau communautaire, avec les bénéficiaires directs de la politique. Le cadre conceptuel de Moore pour les évaluations de processus d'interventions complexes a guidé notre démarche (voir Annexe 1) (28). Les composantes spécifiques du cadre conceptuel de Moore étudiées dans cette évaluation sont le processus (Objectif 1), la fidélité et la portée (Objectif 2) et les résultats (Objectif 3).

L'étude s'est déroulée dans un contexte d'insécurité croissante dans le pays, causée par des attaques terroristes (29). Elle a également été menée peu après qu'une grève nationale ait paralysé les activités non essentielles dans les établissements de santé pendant plusieurs semaines.

**Phase qualitative exploratoire**

En octobre 2019, des documents officiels (manuels de stratégie nationale de planification et de mise en œuvre, guides d'information à l'intention des autorités sanitaires, déclarations de politique générale) ont été collectés afin de recueillir le plus d'informations possible avant de mener des entretiens. En novembre 2019, des entretiens individuels semi-structurés (n=3) ont été menés dans la capitale Ouagadougou avec des planificateurs de programmes au sein du MS impliqués dans l'élaboration de la politique de gratuité de la PF. Les participants ont été sélectionnés de manière opportune avec l'aide d'un courtier de connaissances spécialisé sur les problématiques de santé au Burkina Faso. Les entretiens ont eu lieu dans les bureaux des acteurs du ministère de la Santé. Ils ont été menés en français par LB, ont été supervisés par un chercheur local formé à la recherche qualitative (AB), et ont duré 30-60 minutes. Des notes de terrain ont été prises au cours des entretiens, qui ont fait eux l'objet d'un enregistrement audio. Après trois entretiens, les informations recueillies ont été jugées suffisantes pour permettre de comprendre comment la mise en œuvre de la PF gratuite avait été planifiée. Les données ont été interprétées sur la base de l'examen des documents de planification, des notes de terrain et des enregistrements audio, ainsi que des commentaires des autres membres de l'équipe de recherche. Le fait que de nombreux chercheurs de l'équipe aient été impliqués depuis plusieurs années avec le MS dans des études sur les politiques de gratuité des soins a enrichi cette phase exploratoire.

**Phase de collecte des données quantitatives sur le terrain**

La composante quantitative de cette étude était intégrée dans un autre projet de recherche en cours qui visait à évaluer les impacts de la politique nationale qui a supprimé en 2016 le paiement direct dans les centres de santé pour les femmes enceintes et les enfants de moins de cinq ans. Des questions spécifiques à la politique de gratuité de la PF ont été ajoutées à l'enquête initiale. La composante quantitative a été principalement conçue pour poursuivre l'objectif n°2 de cette étude, à savoir : évaluer la couverture et le niveau de mise en œuvre de la politique de gratuité de la PF au sein de la population cible. La dimension de couverture a été explorée en évaluant la connaissance des bénéficiaires de la politique de PF et de ses facteurs associés, tandis que le niveau de mise en œuvre a été examiné en considérant la présence de coûts résiduels liés à la PF.

**Échantillonnage**

Les procédures d'échantillonnage ont été dérivées de celles du programme d'enquêtes démographiques et sanitaires de l'USAID. Un échantillonnage en grappes a été effectué dans quatre des dix districts sanitaires : Léo et Tenado (Centre-Ouest) et Sindou et Banfora (Cascades). Ces districts ont été sélectionnés à dessein sur la base de deux critères : ils comprenaient des établissements de santé situés en zone rurale, et ils étaient sécuritaires (n'ayant pas connu d'attaques depuis 2016). En utilisant les zones de dénombrement définies par le programme d'enquêtes démographiques et sanitaires dans ces quatre districts, 29 ont été sélectionnées aléatoirement avec une probabilité proportionnelle à la taille de leur population. Dans un deuxième temps, 24 ménages par unité ont été sélectionnés aléatoirement avec une probabilité égale.

La taille cible de l'échantillon de ménages était de 696. Seuls les ménages comptant ≥1 femme âgée de 15 à 49 ans étaient éligibles. Les ménages inéligibles et ceux qui n'ont pu être trouvés ont été remplacés par le ménage éligible avoisinant plus proche.

**Collecte des données**

L'enquête a eu lieu en mars 2020, après une formation de cinq jours pour les enquêteurs. Un questionnaire adapté de celui standardisé utilisé lors des enquêtes démographiques et sanitaires a été administré à toutes les femmes consentantes âgées de 15 à 49 ans des ménages sélectionnés. Il a été administré dans la langue locale par des enquêtrices ayant une expérience préalable en recherche communautaire. Bien que le questionnaire standardisé couvrait déjà les caractéristiques sociodémographiques des participants et l'utilisation des services de PF, certaines questions ont été ajoutées pour enregistrer les déboursements pour les services de PF (normalement couverts désormais par la politique) et la connaissance des participants de la politique de gratuité de la PF.

Les réponses ont été recueillies électroniquement sur des tablettes à l'aide du logiciel Commcare (Dimagi, Cambridge, USA). Les données des questionnaires ont été automatiquement téléchargées sur un serveur sécurisé, puis extraites et nettoyées à l'aide de Stata 14.0 (StataCorp, College Station, TX).

**Analyse**

Une analyse descriptive a été réalisée sur trois variables clés liées à la mise en œuvre de la politique de PF gratuite. La couverture de cette politique a d'abord été évaluée en estimant la proportion de femmes ciblées qui savent que la PF est désormais officiellement gratuite dans les établissements de santé. Deuxièmement, l'utilisation des contraceptifs (et le moment de leur obtention) a été mesurée en classant les femmes selon leur utilisation actuelle des contraceptifs (oui/non) et le moment où elles se les sont procurés pour la dernière fois, c'est-à-dire avant ou après la mise en œuvre de la politique. Troisièmement, les coûts associés à la PF lors de la dernière visite de l'enquêtée dans un établissement de santé ont été analysés selon le moment de la visite (avant / après l'introduction de la politique de gratuité) et ventilés par type de service.

Toutes les analyses statistiques ont été effectuées à l'aide du logiciel libre de statistique R V3.5.2. Les cartes ont été produites à l'aide du logiciel libre QGIS v3.8.1 Zanzibar. Un modèle de régression logistique multivarié avec des estimateurs de variance robustes a été utilisé pour étudier les facteurs liés à la connaissance de la politique de PF. La différence dans la proportion de participants ayant payé pour des services de PF avant et après l'introduction de la politique de gratuité a été estimée par des tests de Chi-deux d'homogénéité de la variance. Le seuil de signification statistique a été fixé à 0,05 (tests bilatéraux).

**Phase de collecte des données qualitatives sur le terrain**

*Échantillonnage*

La composante qualitative consistait en des entretiens individuels semi-dirigés avec le personnel de santé et les femmes membres de la communauté. Pour des raisons de faisabilité et de logistique, la recherche qualitative s'est déroulée uniquement dans le district de Banfora, où cinq centres de santé publics ont été sélectionnés en fonction de leur localisation (zones rurales accessibles) et de leur type (les centres de santé sans service de maternité ont été exclus). Dans chaque centre de santé, l'infirmier chef de poste et un autre membre du personnel de santé (de préférence une sage-femme) ont été interviewés individuellement (n=10). Avec leur aide, des ménages ou groupes de ménages avec des femmes en âge de procréer ont été identifiés dans la zone de recrutement, et deux femmes ont été sélectionnées dans la communauté entourant chaque centre de santé (n=10). La sélection était stratifiée par âge, avec une femme membre de la communauté ayant moins de 20 ans et une autre ayant plus de 20 ans ; ces participantes provenaient de ménages différents. Pour être sélectionnées, les femmes de la communauté devaient utiliser la PF.

*Collecte des données*

Les données ont été collectées en janvier 2020. Pour les membres de la communauté, les entretiens ont eu lieu en dehors de leur domicile, dans un endroit isolé qui garantissait la confidentialité des répondants. Pour le personnel de santé, les entretiens ont eu lieu dans une salle privée de l'établissement de santé. Un guide d'entretien spécifique au type de participant et comportant des questions ouvertes a été utilisé pendant la discussion (voir annexe). Les entretiens ont été menés en français ou en djoula (selon la préférence du participant) par une seule chercheuse ayant reçu une formation approfondie en recherche qualitative.

Les entretiens avec le personnel de santé et les femmes ont duré respectivement 30-50 minutes et 15-30 minutes. Ils ont été enregistrés, retranscrits mot à mot et traduits en français par un assistant. Le chercheur de terrain a écouté les enregistrements audio originaux et a validé les retranscriptions. Dix entretiens ont été réalisés avec chaque type de participant et, grâce aux commentaires de la chercheuse et à ses notes de terrain, la saturation des données a été considérée comme atteinte.

*Analyses*

Les données qualitatives ont fait l'objet d'une analyse de contenu. Les transcriptions ont été lues plusieurs fois pour une compréhension approfondie. Le texte a été entièrement segmenté et un codage mixte inductif et déductif a été utilisé : déductif parce que la grille de codage a d'abord été établie sur la base du cadre conceptuel de Moore, et inductif parce que de nouveaux codes ont été créés pour les thèmes émergents (28). Les concepts du cadre qui ont été utilisés dans la grille de codage déductif étaient les suivants : processus (obstacles et facilitateurs de la mise en œuvre), fidélité (paiement des services ou des méthodes de PF), portée (connaissance de l'intervention) et résultats (qualité perçue des soins) (28). Un double codage a été effectué par deux auteurs sur un échantillon du matériel afin de confirmer la fiabilité de la grille de codage finale. Le codage a été effectué par LB et CB, ainsi que l'analyse thématique. Un débriefing entre pairs a été effectué avec les membres de l'équipe de recherche afin de dégager des thèmes à partir des codes. Les résultats des entretiens ont été triangulés avec les données recueillies lors de la phase exploratoire qualitative. L'analyse a été effectuée à l'aide du logiciel QDA Miner (QDA Miner 5.0).

**Convergence entre les résultats qualitatifs et quantitatifs**

L'intégration des données qualitatives et quantitatives pour la conception convergente a été réalisée spécifiquement pour l'objectif 2 de notre étude : évaluer le niveau de couverture et de mise en œuvre de la politique de gratuité de la PF auprès de la population cible. Pour cet objectif, les résultats quantitatifs et qualitatifs ont été analysés en parallèle pour mieux appréhender l’objet à l’étude, avant d'être intégrés. Les perspectives obtenues à partir des composantes qualitatives et quantitatives ont été comparées afin d’identifier et d’interpréter les divergences et convergences (25). Cette interprétation a été effectuée pour élargir notre compréhension des conclusions sur la politique de gratuité de la PF. En particulier, les résultats qualitatifs ont été utilisés pour confirmer notre conclusion quantitative et expliquer des résultats quantitatifs spécifiques (dépenses pour la PF et niveau de connaissances de la nouvelle politique).

**Considérations d'ordre éthique**

Tous les participants ont donné leur consentement éclairé par écrit pour la collecte des données qualitatives et quantitatives. Pour la phase quantitative, le consentement a été enregistré sur la tablette où le questionnaire a été réalisé. Le questionnaire et les entretiens ont été administrés individuellement dans un endroit isolé afin de préserver la confidentialité des participants. Les participants âgés de 15 à 17 ans ont été considérés comme des mineurs matures et ont donné leur consentement en tant qu'adultes. Toutes les procédures de l'étude, y compris celles relatives au recueil du consentement, ont été approuvées par le Comité d'éthique de la recherche en sciences de la santé de l'Université de Montréal (Certificat #CERSES-20-146-D) et par le Comité d'éthique pour la Recherche en Santé du Burkina Faso (Délibération #2018-6-075). L’organisme ayant financé cette étude n'a joué aucun rôle dans la conception de l'étude, la collecte des données, l'analyse des données, l'interprétation des données et la rédaction du manuscrit.

**Résultats**

Vingt-trois personnes ont participé au volet qualitatif de l'étude : trois agents du ministère de la Santé chargés de la mise en œuvre de la gratuité de la PF au niveau central, 10 agents sanitaires travaillant dans des centres de santé ruraux et 10 femmes membres de la communauté (FCM) des environs. Les agents sanitaires avaient entre 1 et 15 ans d'expérience professionnelle. La moitié des femmes membres de la communauté étaient âgées de 20 à 45 ans ; elles étaient femmes au foyer, agricultrices ou commerçantes. L'autre moitié des FCM était composée d'étudiantes toutes âgées de 19 ans. Bien qu'éligible, aucune FCM âgée de 15 à 18 ans n'a été recrutée.

Au total, 901 femmes en âge de procréer ont participé à la composante quantitative et ont répondu à l'enquête. Leurs principales caractéristiques sociodémographiques sont présentées dans le tableau 1.

**Obstacles à la mise en œuvre**

Différents types d'obstacles ont été mentionnés au cours des entretiens, selon les participants. Tout d'abord, les acteurs du ministère de la Santé ont perçu d'importants obstacles à la mise en œuvre de la nouvelle politique, qu’ils soient structurels ou contextuels. Certains d'entre eux étaient anticipés, comme l'opposition des dirigeants catholiques et des associations religieuses. Ils ont également perçu le risque que la gratuité de la PF soit interprétée à tort comme un encouragement à l'avortement, ce qui aurait suscité la résistance du public. D'autres obstacles étaient contextuels, comme le climat d'insécurité dans le pays qui a entraîné une diminution de l'accès de la population aux établissements de santé où les services de PF sont offerts et, vice versa, une diminution de l'accès du personnel de santé aux communautés. De plus, la grève du personnel de santé en 2019 a encore limité le nombre d'activités communautaires réalisées par celui-ci et a entravé le plan de communication entourant la PF. Ces problèmes ont justifié des ajustements dans la planification de la politique, notamment en réduisant l'intensité de la stratégie de communication. Les acteurs du MS ont reconnu que, dans ce contexte particulier, ils ont décidé de faire profil bas pour éviter les protestations. Les acteurs du ministère de la Santé ont estimé qu'il y avait peu de communication officielle du niveau central vers la population et qu'ils ne s'attendaient pas à ce que la politique ait un effet significatif sur l'utilisation de la PF.

*"Les gens confondent souvent PF et avortement [...]. C'est cette confusion qui nous crée souvent des problèmes. Car dès que nous avons adopté la politique de gratuité en décembre [...], nous avons été appelés par l'Église catholique. J'ai dû aller à la cathédrale pour expliquer le problème lié à la PF libre et donner des précisions "* (MS#1)

*''Vous voyez, c'est parce que la communication n'a pas suivi. Même vous au niveau central, vous n'avez pas entendu grand-chose. Parce que logiquement on aurait dû communiquer là-dessus sur toutes les radios, on aurait dû parler''* (MS#2).

*''Parce que les gens ont besoin d'avoir l'information que c'est gratuit. Mais la communication était plus occupée à gérer la grève. Donc j'ai peur que les résultats soient atténués, qu'on n'ait pas vraiment de différence entre la période avant la politique de PF gratuite et la période pendant la politique de PF gratuite''* (MS#2).

Le personnel de santé a désigné les ruptures de stock comme l'obstacle le plus important à la mise en œuvre. Ces pénuries concernaient principalement les seringues, mais aussi les gants, les compresses, le ruban adhésif médical, les pinces à épiler et les scalpels pour retirer les implants. Les femmes ont confirmé avoir vécu des situations où elles ont dû payer pour se procurer certains consommables en rupture de stock dans la structure sanitaire.

*''C'était la pénurie de seringues. (...) Si quelqu'un vient pour une méthode d'insertion d'implant, il n'y a pas de seringues. Donc ils doivent aller en acheter ailleurs''* (PS#3).

*'' Parfois il n'y a plus de gants, il faut aller en acheter''* (FMC#5, 45 ans).

Ces pénuries auraient été déclenchées par l'augmentation rapide de la demande de services de PF et de contraceptifs après l'introduction de la politique. Quelques femmes ont également mentionné qu'elles avaient été confrontées à des ruptures de stock pour leur méthode contraceptive, bien que la plupart des prestataires aient indiqué que ces pénuries étaient moins fréquentes que celles des consommables. Un autre facteur contributif est la lenteur du mécanisme de remboursement. En effet, les établissements de santé doivent absorber les coûts des services de PF et demander ensuite à être remboursés par le gouvernement ; cependant, ils subissent parfois un retard de plusieurs mois avant de recevoir le remboursement, ce qui les met sous pression financière et contribue aux pénuries de stocks.

*"Il est difficile d'être remboursé, ce qui signifie qu'il n'est pas facile d'obtenir de l'argent pour acheter des fournitures. Parce que si on ne vous rembourse pas, on ne peut pas acheter de fournitures"* (PS#1).

Ces pénuries sont perçues comme préjudiciables. Non seulement elles réduisent l'accès aux contraceptifs gratuits, mais elles découragent également les bénéficiaires de les utiliser systématiquement. Des tensions locales et la méfiance des femmes envers le personnel de santé peuvent également résulter de ces situations.

*"Pour la PF, vous serez dehors au moins trois ou quatre jours avant d'aller passer une commande. Ces trois jours, ce n'est pas rien. Je prends un vendredi comme ça, parce que c'est le jour du marché le vendredi, beaucoup de femmes vont venir, s'il se trouve que vous n'avez plus le produit, vous savez ? Donc l'intervalle de trois, cinq jours comme ça, avant d'aller faire la prochaine commande, surtout en termes de PF, en tout cas ça se ressent. Et puis ça décourage"* (PS#2).

**Facteurs favorables à la mise en œuvre**

Plusieurs éléments ont été favorables à la mise en œuvre de la nouvelle politique. Le plus important est qu'une fois par an, depuis 2012, il y a une initiative appelée « Semaine nationale de la PF » au Burkina Faso. Durant ces semaines spécifiques, tous les services de PF étaient déjà offerts gratuitement. Cette initiative a été décrite, tant au niveau central que périphérique, comme une opportunité pour la population et le personnel de santé de se familiariser avec la future politique de gratuité de la PF.

Un autre facilitateur clé découle de la stratégie de mise à l'échelle fonctionnelle. En effet, avant la politique de gratuité de la PF, les frais d'utilisation avaient déjà été supprimés dans tous les établissements de santé publique pour les femmes enceintes et les enfants de moins de cinq ans. La mise en œuvre de cette nouvelle politique a été facilitée puisque, d'un point de vue institutionnel, de nouveaux services ont simplement été ajoutés au panier de services gratuits.

Contrairement à l'hypothèse formulée par les planificateurs de programmes du ministère de la Santé au niveau central, selon laquelle la résistance de la population à la PF aurait constitué un obstacle, les participants (FMC et PS) étaient favorables à la politique de gratuité de la PF. Les FMC ont déclaré que la PF leur permet de se reposer en augmentant l'espacement des naissances, et que la nouvelle politique est particulièrement utile pour les femmes ayant des moyens financiers limités. Les membres du personnel de santé sont d'accord et confirment qu'ils soutiennent la politique parce qu'elle aide les femmes dans le besoin et a des effets positifs sur la santé.

Certains participants ont également mentionné que la PF n'est plus taboue, et que les membres de la communauté, y compris les hommes, la soutiennent de plus en plus.

*''Je pense que les hommes ont compris. Ils commencent à donner le feu vert aux dames pour venir mettre la PF''* (PS#3).

*''Je vois que ça aide les femmes, nous qui sommes aussi à l'école, ça nous aide à poursuivre nos études, nos activités professionnelles''* (FMC#2, 19 ans).

Selon les agents de santé, la résistance plus faible que prévu de la population peut être en partie attribuée aux activités de sensibilisation de plusieurs organisations non gouvernementales (ONG) du district, telles que Pathfinder, Marie Stopes International et Ma Copine. Ces ONG auraient également facilité la mise en œuvre de la nouvelle politique en soulageant le personnel de santé de certaines activités communautaires de sensibilisation à la PF.

**Connaissance de la politique de gratuité de la PF**

Dans l'ensemble, la moitié (50,4%) des participantes à l'enquête connaissaient la nouvelle politique. La probabilité de connaître l'existence de la nouvelle politique différait significativement entre les districts, même après ajustement d'un ensemble de variables de confusion potentielles au niveau des individus et des ménages (tableau 2). Par rapport au district le plus peuplé (Banfora), le rapport de cotes était plus faible à Sindou (aOR = 0,41, IC 95% [0,23-0,71]), et plus élevé à Leo (aOR = 2,04, IC 95% [1,36-3,04]) et Tenado (aOR = 3,38, IC 95% [1,75-6,50]). Les autres variables de nature environnementale ou géographique (c'est-à-dire le milieu urbain ou rural et la distance entre le ménage et le centre de santé le plus proche) n'étaient pas significativement associées à la connaissance de la nouvelle politique.

Les entretiens ont confirmé que la majorité des femmes membres de la communauté n'avaient pas été informées des nouveaux services gratuits offerts. De plus, parmi celles qui le savaient, les femmes ont mentionné ne pas avoir suffisamment d'informations sur ce qu'impliquait la gratuité de la PF, notamment sur les services inclus et si les effets secondaires de la PF étaient couverts par la politique.

*"Je n'ai pas assez d'informations à ce sujet [politique de PF gratuite]. Je sais seulement que les méthodes de PF sont gratuites. L'agent de santé n'a rien dit de plus"* (FMC#1, 20 ans)

*"Je veux plus d'informations sur la PF gratuite ou si par exemple j'utilise une méthode et qu'il m'arrive quelque chose en conséquence, comment cela fonctionne-t-il ?"* (FMC#5, 45 ans)

Outre le lieu, certaines caractéristiques sociodémographiques étaient également associées à une probabilité accrue de connaître la nouvelle politique (tableau 2). En particulier, le modèle suggère une probabilité accrue de connaître la nouvelle politique si les femmes sont en couple ou sexuellement actives, si elles ont reçu une éducation primaire, si elles ont vu un agent de santé au cours des 12 derniers mois et si leur ménage possède une radio.

L'importance de la radio pour recevoir des informations sur la politique de gratuité a également été mentionnée lors des entretiens. De nombreux participants ont déclaré avoir entendu parler de la politique de gratuité de la PF par la radio.

*" [...] c'est la radio locale ici qui nous aide vraiment, qui est vraiment écoutée par la population, qui nous aide dans les messages qu'elle diffuse. ''* (PS#3)

*"J'ai entendu parler de ça à la radio".* (FMC#2, 19 ans)

Certaines femmes ont également déclaré avoir reçu l'information par leur centre de santé ou par des agents de santé. La transmission de l'information dans la communauté par le bouche-à-oreille ou par des animateurs de village a également été fréquemment mentionnée.

*"Je suis allée au centre de santé et les agents de santé nous ont informés"* (FMC#2, 38 ans).

*"Les gens de ma communauté m'ont informé"* (FMC#1, 19 ans).

*"Je pense que l'information a aussi circulé par le bouche-à-oreille"* (PS#5).

Enfin, après ajustement sur l'éducation et l'état civil, le modèle n'a montré aucune association entre la connaissance de la nouvelle politique et l'âge des participants. Cependant, les entretiens avec les adolescentes ont révélé qu'elles étaient particulièrement mal informées : seule une adolescente sur les cinq interrogées a été informée, dans son école, de la nouvelle politique de gratuité pour la PF.

*"Ils nous ont donné l'information par le biais de notre école. C'est même un médecin qui a donné l'information"* (PS#3, 19 ans).

**Coût des visites de PF**

Environ 66% (127/191) des participantes à l'enquête qui ont obtenu leur dernière méthode contraceptive avant l'introduction de la PF gratuite ont dû la payer. Cette proportion a été réduite à 39% (45/115) pour les femmes qui ont payé leur dernière méthode contraceptive après l'introduction de la nouvelle politique (changement : -27%, IC 95% [-15,5 à -39,2]). Cette réduction était trois fois plus importante dans la région des Cascades que dans la région du Centre-Ouest (tableau 3).

Les acteurs du ministère de la Santé et les travailleurs de la santé ont déclaré lors des entretiens que toutes les méthodes et services de PF étaient maintenant officiellement offerts gratuitement dans leurs établissements de santé. Cependant, à l'instar de ce qui a été observé dans l'enquête, les femmes interrogées ont mentionné qu'elles devaient toujours payer pour la PF, même si elle est officiellement gratuite.

*"C'est en fait, c'est vraiment gratuit. Quand les femmes viennent, elles choisissent simplement leur méthode"* (PS#3).

*"J'en ai entendu parler à la radio. Mais quand je suis allée au centre de santé pour ma PF, ce n'était de toute façon pas gratuit* [alors qu’elle aurait dû l’être]*..."* (FMC#2, 19 ans).

Les femmes ont donné plus d'informations sur cette situation et ont rapporté que, malgré la politique, ce sont surtout les consommables qui demeurent payants, et parfois les contraceptifs eux-mêmes. Les agents de santé ont reconnu ce fait en expliquant que la pénurie de consommables ou de contraceptifs dans les centres de santé obligeait les femmes à les acheter ailleurs. Les données de l'enquête confirment ce constat ; après l'introduction de la politique, les coûts résiduels liés à la PF concernaient principalement la méthode contraceptive elle-même, et non la consultation (annexe 3).

*"Mais souvent on est même obligé de se fournir dans les pharmacies de la ville. C'est un peu compliqué parce que [...] on ne peut pas donner ça aux clients gratuitement. Ils doivent payer le prix du pharmacien"* (PS#10).

*"Quand ils [les agents de santé] disent qu'ils n'ont pas ce matériel, ils vous disent de l'acheter ailleurs"* (FMC#2, 38 ans).

**Qualité perçue des services de PF**

Dans l'ensemble, les femmes sont satisfaites de la qualité des services de PF et ont mentionné avoir eu des expériences positives avec les agents de santé.

*"J'ai vu que dans de nombreux centres de santé, les agents de santé s'occupent bien des femmes. Je ne vais pas mentir. Il y a même un monsieur, il connaît son travail. Il explique bien l'utilisation des contraceptifs"* (FMC#3, 40 ans).

*"Le jour où j'y suis allée, quand mon tour est arrivé, je suis entrée seule. Ils m'ont donné de bons conseils. Ils m'ont tout expliqué"* (FMC#2, 19 ans).

Cependant, certains problèmes d'accès auraient atténué la qualité perçue des soins, notamment les rendez-vous reportés et les longues périodes d'attente. Les agents de santé ont reconnu ces problèmes qui, selon eux, sont attribuables à l'augmentation de la charge de travail. Ils ont admis être dépassés par la forte demande de services de PF, ce qui les a obligés à augmenter le rythme des consultations avec les femmes. Cette charge de travail accrue a pu affecter les relations entre les patientes et les prestataires.

*"D'autres peuvent venir le matin et les agents de santé ont beaucoup à faire, alors ils peuvent demander à revenir le soir. D'autres viennent aussi sans succès et ce n'est que le lendemain qu'elles parviennent à obtenir leur contraceptif"* (FMC#5, 45 ans).

*"J'ai attendu longtemps. Je suis allée le matin vers 6 heures. Quand je suis revenue, il était 13 heures"* (FMC#2, 19 ans)

*"Je ne peux pas vous mentir, on n'a pas le temps de passer presque [une heure] avec un patient, oh non, c'est un peu difficile. Mais dire qu'on va prendre du temps avec un patient comme on le faisait avant, ça va être un peu compliqué"* (PS#5).

*"La PF gratuite est arrivée et le taux d'utilisation de l'implant a triplé [...]. Les femmes qui adhèrent à la PF ont doublé pendant la PF gratuite [...]. Votre humeur va aussi changer, et votre accueil ne sera pas le même. La communication avec les patientes n'est pas la même. Donc, cela a aussi une influence négative"* (PS#2)

Dans certains établissements, les travailleurs sociaux ont également adapté leurs pratiques cliniques en organisant des séances de groupe plutôt que des consultations individuelles. Par exemple, il arrive que les femmes soient rassemblées pour un conseil en PF.

*"Oui, oui, s'il y en a beaucoup comme ça [plusieurs femmes à consulter], on fait du counseling de groupe en ce moment"* (PS#8).

**Discussion**

À notre connaissance, cette étude est la première à explorer le processus de mise en œuvre d'une politique de gratuité de la PF en ASS, à évaluer la connaissance de la politique parmi la population rurale cible après six mois, et à étudier son influence sur la qualité perçue des services de PF. Bien que la mise en œuvre de politiques de gratuité des soins ait déjà été examinée dans différents contextes, cette étude a identifié de nouveaux facteurs d’influence qui semblent être plus spécifiques à la suppression des frais d'utilisation pour les services de PF.

Notre étude est conforme à la prédiction selon laquelle il y aurait une augmentation de la demande de services de PF et un meilleur accès à ces services après la suppression des frais d'utilisation. Comme cela a été observé dans de nombreux contextes où des politiques de gratuité des soins ont été introduites (7, 8, 14, 17), cette augmentation immédiate a généré des obstacles à la mise en œuvre : pénuries de médicaments, retards dans la distribution des consommables et perception d'une charge de travail accrue par les agents de santé. Ces obstacles ont fait en sorte que les femmes devaient encore payer (partiellement) pour les services de PF après l'introduction de la nouvelle politique, bien que moins souvent qu’auparavant. Des problèmes similaires ont été observés après la suppression des frais d'utilisation pour les femmes enceintes et les enfants ; les preuves recueillies ailleurs suggèrent que ces problèmes sont transitoires et tendent à diminuer progressivement dans des conditions de mise en œuvre routinière (12, 13, 17). Sur la voie de la routinisation, le problème du financement durable ne doit pas être ignoré ; cependant, cette politique est considérée comme une priorité nationale et est directement imputable au budget régulier de l’État (23).

Si la suppression des frais d'utilisation est généralement bien accueillie par les utilisateurs, les planificateurs politiques s'attendaient à une certaine résistance de la part des associations religieuses et de la population générale dans le cas des services de PF, notamment parce qu'ils sont parfois confondus avec l'avortement. Nos résultats ne confirment pas cette prédiction et montrent que tant les femmes que les travailleurs de santé sont favorables à la nouvelle politique. Les participants ont rapporté qu'il y avait une certaine opposition à la PF dans la population générale (maris, chefs religieux, etc.) mais aucune résistance à la nouvelle politique en soi. Trois facteurs clés ont facilité la mise en œuvre de la nouvelle politique, notamment en améliorant son acceptabilité : l'expérience antérieure de la semaine nationale de gratuité de la PF, l'implication des groupes de femmes et des ONG, et le fait que la plupart des procédures administratives étaient déjà en place (puisqu'il s'agissait d'une extension fonctionnelle).

Bien que la politique semble être largement acceptée, il convient d'être prudent avant de tirer des conclusions. En effet, la politique a facilité l'accès aux services de PF, mais les preuves scientifiques suggèrent que les politiques de suppression des frais d'utilisation n’entraînent pas nécessairement à elles seules une autonomie décisionnelle accrue des femmes en matière de PF (30). Une autre étude menée au Burkina Faso après la suppression des frais d'utilisation des services de PF a signalé une augmentation des tensions conjugales (31). Dans certains cas, les prestataires de soins ont dû adapter leurs pratiques pour garantir la confidentialité et la sécurité des femmes (31).

Notre analyse indique que la connaissance de la nouvelle politique était faible parmi les femmes en âge de procréer (~50%), même six mois après son introduction. Après plus de 10 ans d'expérience de recherche sur les politiques de gratuité des soins au Burkina Faso, c'est la première fois que nous observons cela. L'information sur les politiques qui introduisent ou suppriment les frais d'utilisation se diffuse généralement rapidement, bien que les mauvaises interprétations et la connaissance imparfaite des détails de la politique soient courantes (32-34). La situation observée est probablement due à la décision des autorités sanitaires de ne pas diffuser largement l'information selon laquelle la PF était devenue gratuite, par crainte de la résistance de la communauté. Les analyses qualitatives et quantitatives ont permis d'identifier plusieurs facteurs qui étaient positivement associés à la connaissance de la nouvelle politique : possession d'une radio, visite récente dans un établissement de santé ou visite à domicile d’un professionnel de santé, niveau d'éducation, et être marié ou sexuellement actif. Même si l'information n'a pas été officiellement diffusée à la radio, elle a néanmoins circulé sur les radios communautaires, ce qui s'est avéré être une stratégie de diffusion efficace dans les pays d'Afrique de l'Ouest (35, 36). Plusieurs stratégies complémentaires pourraient être utilisées non seulement pour augmenter le nombre de personnes qui connaissent la politique, mais aussi pour engager le dialogue avec les communautés sur la PF. Ces stratégies potentielles comprennent, entre autres, des groupes de discussion communautaire, des réunions avec les chefs de village et les agents de santé communautaires, l'utilisation de "crieurs publics" et les messages textuels / audio sur les téléphones cellulaires (37). Il importe que ces stratégies soient adaptées à la population ciblée et à son milieu de vie (38, 39).

Les femmes les plus informées sur la politique avaient tendance à avoir un niveau d'éducation plus élevé et à avoir des contacts plus fréquents avec le centre de santé. Les résultats suggèrent également, bien que cela ne soit pas statistiquement significatif, qu'il existe un gradient avec le statut socio-économique. Cela signifie que les avantages de la politique de gratuité dans les communautés rurales iront sans doute d'abord aux femmes plus privilégiées avant d'atteindre celles qui sont moins bien loties, ce qui pourrait augmenter les inégalités en matière de santé (40, 41). Des manifestations similaires de l'hypothèse de l'équité inverse ont été signalées après la suppression du paiement direct pour les accouchements par césarienne au Bénin et au Mali (42). Ceci est particulièrement problématique dans le cas de la PF, puisque les besoins non satisfaits pour ces services sont connus pour être plus élevés chez les femmes moins bien nanties (43, 44).

Dans le même ordre d'idées, nos résultats indiquent que la connaissance de la nouvelle politique était plus faible chez les adolescentes et les femmes qui n'étaient pas encore sexuellement actives. Pendant des décennies, les interventions en matière de PF en Afrique subsaharienne se sont principalement concentrées sur les femmes adultes et mariées. De nombreux appels à s'écarter de ce modèle ont conduit à conceptualiser la santé des femmes dans un cadre basé sur les droits humains (45, 46). En théorie, la suppression des frais d'utilisation des services de PF s'inscrit dans un tel cadre puisqu'elle augmente l'accès financier en utilisant une approche populationnelle (qui concerne tout le monde de manière égale). En pratique, les adolescentes et les femmes célibataires risquent d'être discriminées dans leur droit d'accès à la contraception, avec de nombreuses répercussions négatives sur leur santé (47). Il est essentiel de s'attaquer à ce problème pour promouvoir le droit des femmes à la santé reproductive de manière équitable, ce qui est une obligation pour les gouvernements.

Malgré l'utilisation d'une approche riche basée sur des méthodes mixtes pour déterminer et comprendre le niveau de mise en œuvre de la politique de gratuité de la PF, cette étude présente certaines limites. Alors que les données quantitatives ont été recueillies dans quatre districts, les données qualitatives n'ont été recueillies que dans un seul. Ceci a rendu impossible l'intégration des données quantitatives et qualitatives pour les données désagrégées par district. Nos résultats et interprétations peuvent ne pas être représentatifs des processus de mise en œuvre dans d'autres zones où l’initiative pilote a été introduite. Il n'a pas été possible d'interviewer des jeunes femmes (15-18 ans) qui utilisaient la contraception, ce qui empêche une compréhension approfondie de leurs perceptions de la politique. De plus, même si elles ne nous ont pas été adressées individuellement, les femmes de la communauté ont été recrutées pour un entretien avec l'aide du personnel de santé, ce qui comporte un risque de biais de sélection. Pour des raisons logistiques, cette étude s'est concentrée sur la qualité perçue des services de PF plutôt que d'utiliser une méthode objective d'évaluation de la qualité. La combinaison des enquêtes et des entretiens avec une observation directe aurait pu produire une meilleure représentation de la qualité. Un effet Hawthorne ne peut être exclu et il est possible qu'un biais de désirabilité sociale ait affecté les réponses des participants sur des sujets sensibles (48). Pour réduire ce risque, les entretiens individuels ont été réalisés par des femmes hautement formées aux méthodes de recherche qualitative. Un biais de rappel est également plausible, notamment pour les questions concernant les pratiques des femmes avant la mise en œuvre de la politique. Une façon potentielle de limiter ce biais aurait été de réaliser des entretiens de référence avant la mise en œuvre de la politique. Cependant, l'étude a eu lieu le plus rapidement possible (six mois) après l'introduction de la politique afin de réduire ce biais potentiel.

**Recommandations**

Depuis 2016, le ministère de la Santé du Burkina Faso a successivement supprimé les frais d'utilisation pour les enfants de moins de cinq ans, pour les soins de santé maternelle et reproductive, et maintenant pour les services de PF, dans tous les établissements de santé publics. Les effets positifs de ces initiatives sur l'amélioration de la santé maternelle et infantile et la réduction des inégalités de santé doivent être soulignés. Sur la base des résultats de la présente étude, il semble particulièrement important d'accroître la sensibilisation ou la connaissance de la politique de PF parmi les groupes vulnérables tels que les adolescentes, les femmes célibataires et les femmes de statut socio-économique inférieur. Ces stratégies devraient aller au-delà de la diffusion de messages à la radio, de manière officielle ou informelle. Il semble également que des idées fausses persistent sur la PF, qui est encore parfois confondue avec l'avortement. Mais ces idées fausses ne devraient pas empêcher les efforts pour promouvoir le droit des femmes à la santé sexuelle et reproductive. La mise en œuvre réussie de cette politique au Burkina Faso montre que, tout en combattant ces idées fausses, il est possible de promouvoir le droit des femmes à accéder aux services de planification familiale. Cependant, un meilleur accès ne signifie pas automatiquement une plus grande autonomie décisionnelle, et des mesures complémentaires pour promouvoir l'autonomisation des femmes restent essentielles.

**Conclusion**

Six mois après son introduction en tant que projet pilote dans deux régions du Burkina Faso, la politique d’exemption du paiement direct pour la PF a été confrontée à des obstacles de mise en œuvre qui réduisent probablement son efficacité, notamment des ruptures de stock, des retards dans les remboursements et une communication insuffisante. Il est inquiétant de constater que la connaissance de la nouvelle politique est plus faible chez les adolescentes, les femmes célibataires et les femmes ayant un statut socio-économique inférieur. Il est à espérer que ces obstacles seront transitoires et qu'ils n'aggraveront pas les inégalités en matière de santé. L'acceptabilité de la nouvelle politique a été facilitée grâce aux campagnes de sensibilisation et aux expériences précédentes lors de la semaine annuelle de la gratuité de la PF. Les résultats suggèrent une augmentation du nombre de femmes utilisant les services de PF, tant dans les zones rurales qu'urbaines. Il est remarquable que la nouvelle politique s'inscrive dans la promotion du droit des femmes à la santé reproductive, mais une réflexion plus approfondie est nécessaire pour concilier un meilleur accès aux services de PF et une meilleure autonomisation des femmes.

**Abréviations**

aOR : odds ratio ajusté

CI : Intervalle de confiance

FMC : Femme membre de la communauté

PF : Planification familiale

PS : Personnel de santé

MS : Ministère de la santé

ONG : Organisations non gouvernementales

ASS : Afrique sub-saharienne

**Déclarations**

*Approbation éthique et consentement à la participation*

L'étude a été approuvée par le Comité d'éthique de la recherche en sciences de la santé de l'Université de Montréal (Certificat #CERSES-424 20-146-D) et par le Comité d'éthique pour la Recherche en Santé au Burkina Faso (Délibération #2018-425 6-075). Tous les participants à l'enquête ou aux entretiens ont fourni un consentement éclairé écrit. Le questionnaire a été administré individuellement, ainsi que les entretiens, dans un endroit isolé, afin de préserver la confidentialité des participants.

*Consentement à la publication*

Non applicable.

*Disponibilité des données et du matériel*

Toutes les données anonymisées peuvent être mises à disposition en contactant l'auteur correspondant sous réserve d'une demande raisonnable.

*Conflits d’intérêts*

Les auteurs déclarent qu'ils n'ont pas de conflits d’intérêts.

*Financement*

Cette étude a été réalisée grâce à une subvention de l'initiative Innovating for Maternal and Child Health in Africa - un partenariat d'Affaires mondiales Canada (GAC), des Instituts de recherche en santé du Canada (IRSC) et du Centre de recherches pour le développement international (CRDI) du Canada. Le Réseau pour la Recherche en Santé des Populations du Québec a également fourni un soutien financier. Les organismes subventionnaires n'ont joué aucun rôle dans la conception de l'étude, la collecte et l'analyse des données, l'interprétation des résultats ou la rédaction du manuscrit.

*Contributions des auteurs*

TD et AB1 (Abel Bicaba) ont conçu l'étude. LB, CT, FB et AB2 (Alice Bila) ont collecté les données. TD, LB, SC et CT ont analysé et interprété les données et ont rédigé le premier projet. Tous les auteurs ont effectué une révision critique de l'article et ont approuvé la version finale du manuscrit.

*Remerciements*

Nous remercions les communautés de la zone d'étude qui ont permis la réalisation de ce travail, tous les participants, les autorités sanitaires du district pour leur soutien, et Mme Loula Burton pour la relecture du manuscrit.

**Bibliographie**

1. Robert E, Ridde V. Global health actors no longer in favor of user fees: a documentary study. Global Health. 2013;9(29).

2. Bongaarts J, Cleland J, Townsend J, Bertrand J, Das Gupta M. Family Planning Programs for the 21st Century: Rationale and Design. New York: Population Council; 2012.

3. Korachais C, Macouillard E, Meessen B. How User Fees Influence Contraception in Low and Middle Income Countries: A Systematic Review. Stud Fam Plann. 2016;47(4):341-56.

4. UNICEF. Facts for Life. New York: UNICEF; 2010.

5. Ministère de la Santé du Burkina Faso. Plan National d’Accélération de Planification Familiale du Burkina Faso 2017-2020. Ouagadougou: Direction de la santé de la famille du Ministère de la santé du Burkina Faso; 2017.

6. INSD. Enquête modulaire démographie et santé (EMDS) 2015 du Burkina Faso. Ouagadougou: Institut national des statistiques de la démographie (INSD); 2015.

7. Tran NT, Yameogo WME, Gaffield ME, Langwana F, Kiarie J, Kulimba DM, et al. Postpartum family-planning barriers and catalysts in Burkina Faso and the Democratic Republic of Congo: a multiperspective study. Open Access J Contracept. 2018;9:63-74.

8. Babalola S, John N. Factors underlying the use of long-acting and permanent family planning methods in Nigeria: a qualitative study. Baltimore: Johns Hopkins Bloomberg School of Public Health; 2012.

9. Perkins M, Brazier E, Themmen E, Bassane B, Diallo D, Mutunga A, et al. Out-of-pocket costs for facility-based maternity care in three African countries. Health policy planning. 2009;24(4):289-300.

10. Haddad S, Nougtara A, Fournier P. Learning from health system reforms: lessons from Burkina Faso. Tropical Medicine International Health. 2006;11(12):1889-97.

11. Ridde V, Kouanda S, Bado A, Bado N, Haddad S. Reducing the medical cost of deliveries in Burkina Faso is good for everyone, including the poor. PloS one. 2012;7(3):e33082.

12. Kruk ME, Mbaruku G, Rockers PC, Galea S. User fee exemptions are not enough: out-of-pocket household financing of “free” delivery services in rural Tanzania. Trop Med Int Health. 2008;13(12):1442-51.

13. Asante F, Chikwama C, Daniels A, Armar-Klemesu M. Evaluating the economic outcomes of the policy of fee exemption for maternal delivery care in Ghana. Ghana medical journal. 2007;41(3):110-7.

14. Druetz T, Bicaba A, Some T, Kouanda S, Ly A, Haddad S. Effect of interrupting free healthcare for children: Drawing lessons at the critical moment of national scale-up in Burkina Faso. Social Science Medicine. 2017;185:46-53.

15. Druetz T, Fregonese F, Bado A, Millogo T, Kouanda S, Diabaté S, et al. Abolishing fees at health centers in the context of community case management of malaria: what effects on treatment-seeking practices for febrile children in rural Burkina Faso? PLoS One. 2015;10(10):e0141306.

16. Nguyen HT, Zombré D, Ridde V, De Allegri M. The impact of reducing and eliminating user fees on facility-based delivery: a controlled interrupted time series in Burkina Faso. Health Policy Planning. 2018;33(8):948-56.

17. De Allegri M, Ridde V, Louis VR, Sarker M, Tiendrebéogo J, Yé M, et al. Determinants of utilisation of maternal care services after the reduction of user fees: a case study from rural Burkina Faso. Health policy. 2011;99(3):210-8.

18. Ridde V, Robert E, Meessen B. A literature review of the disruptive effects of user fee exemption policies on health systems. BMC Public Health. 2012;12(289).

19. Mathauer I, Mathivet B, Kutzin J. Les politiques de «gratuité»: opportunités et risques en marche vers la couverture sanitaire universelle. Geneva: World Health Organization; 2017.

20. Béland D, Ridde V. Ideas and policy implementation: Understanding the resistance against free health care in Africa. Global Health Governance. 2016;10(3).

21. Meessen B, Gilson L, Tibouti A. User fee removal in low-income countries: sharing knowledge to support managed implementation. Health Policy and Planning. 2011;26.

22. Witter S, Arhinful D, Kusi A, Zakariah–Akoto S. The Experience of Ghana in Implementing a User Fee Exemption Policy to Provide Free Delivery Care. Reproductive Health Matters. 2007;15(30):61-71.

23. Bicaba F, Browne L, Kadio K, Bila A, Bicaba A, Druetz T. National user fee abolition and health insurance scheme in Burkina Faso: How can they be integrated on the road to universal health coverage without increasing health inequities? Journal of Global Health. 2020;10(1).

24. WHO. Nine steps for developing a scaling-up strategy. Geneva: World Health Organization; 2010.

25. Creswell JW, Clark VLP. Designing and conducting mixed methods research. Thousand Oaks: Sage; 2017.

26. Patton MQ. Qualitative research & evaluation methods : integrating theory and practice. Fourth edition. ed. Thousand Oaks: SAGE; 2015. xxi, 806 pages p.

27. Hamilton AB, Finley EP. Qualitative methods in implementation research: An introduction. Psychiatry Res. 2019;280:112516-.

28. Moore GF, Audrey S, Barker M, Bond L, Bonell C, Hardeman W, et al. Process evaluation of complex interventions: Medical Research Council guidance. BMJ. 2015;350(h1258).

29. Druetz T, Browne L, Bicaba F, Mitchell MI, Bicaba A. Effects of terrorist attacks on access to maternal healthcare services: a national longitudinal study in Burkina Faso. BMJ Global Health. 2020;5(9).

30. Plouffe V, Bicaba F, Bicaba A, Druetz T. User fee policies and women’s empowerment: a systematic scoping review. BMC Health Services Research. 2020;20(1):1-14.

31. Beaujoin C, Bila A, Bicaba F, Plouffe V, Bicaba A, Druetz T. Women’s decision-making power in a context of free reproductive healthcare and family planning in rural Burkina Faso. BMC Women's Health. 2021;21(1):1-11.

32. Druetz T. Integrated primary health care in low-and middle-income countries: a double challenge. BMC Medical Ethics. 2018;19(1):89-96.

33. Druetz T, Bila A, Bicaba F, Tiendrebeogo C, Bicaba A. Free healthcare for some, fee-paying for the rest: adaptive practices and ethical issues in rural communities in the district of Boulsa, Burkina Faso. Global Bioethics. 2021;32(1):100-15.

34. Belaid L, Ridde V. Contextual factors as a key to understanding the heterogeneity of effects of a maternal health policy in Burkina Faso? Health Policy Planning. 2015;30(3):309-21.

35. Bowen H. Making Connections: Using data on Ghanaians’ media use and communication habits as a practical tool in development work. Washington, DC and London: InterMedia Survey Institute; 2010.

36. Yaya S, Uthman OA, Amouzou A, Bishwajit G. Mass media exposure and its impact on malaria prevention behaviour among adult women in sub-Saharan Africa: results from malaria indicator surveys. Global Health Research and Policy. 2018;3(1):20.

37. Djingarey M, Barry R, Bonkoungou M, Tiendrebeogo S, Sebgo R, Kandolo D, et al. Effectively introducing a new meningococcal A conjugate vaccine in Africa: the Burkina Faso experience. Vaccine. 2012;30(B40-B45).

38. Druetz T, Ridde V, Kouanda S, Ly A, Diabaté S, Haddad S. Utilization of community health workers for malaria treatment: results from a three-year panel study in the districts of Kaya and Zorgho, Burkina Faso. Malaria Journal. 2015;14(1):1-12.

39. Arnaert A, Ponzoni N, Debe Z, Meda MM, Nana NG, Arnaert S. Experiences of women receiving mhealth-supported antenatal care in the village from community health workers in rural Burkina Faso, Africa. Digital Health. 2019;5(2055207619892756).

40. Rose G. Sick individuals and sick populations. International Journal of Epidemiology. 2001;30(3):427-32.

41. Frohlich KL, Potvin L. Transcending the known in public health practice: the inequality paradox: the population approach and vulnerable populations. American Journal of Public Health. 2008;98(2):216-21.

42. Ravit M, Audibert M, Ridde V, De Loenzien M, Schantz C, Dumont A. Removing user fees to improve access to caesarean delivery: a quasi-experimental evaluation in western Africa. BMJ Global Health. 2018;3(1):e000558.

43. Victora CG, Vaughan JP, Barros FC, Silva AC, Tomasi E. Explaining trends in inequities: evidence from Brazilian child health studies. The Lancet. 2000;356(9235):1093-8.

44. Maïga A, Hounton S, Amouzou A, Akinyemi A, Shiferaw S, Baya B, et al. Trends and patterns of modern contraceptive use and relationships with high-risk births and child mortality in Burkina Faso. Global Health Action. 2015;8(1):29736.

45. UN-HCHR. Summary Reflection Guide on a Human Rights- Based Approach to Health. Geneva: Office of the High Commission of Human Rights; 2015.

46. Cottingham J, Germain A, Hunt P. Use of human rights to meet the unmet need for family planning. The Lancet. 2012;380(9837):172-80.

47. Oluseye A, Waterhouse P, Hoggart L. ‘I really wanted to abort’Desire for abortion, failed abortion and forced motherhood in South-Western Nigeria. Global Public Health. 2021;20:1-14.

48. Miles MB, Huberman AM, Saldana J. Qualitative data analysis: A methods sourcebook. Thousand Oaks: Sage; 2014.

**Légendes**

**Figures**

Figure 1 Carte de la zone à l’étude.

Les quatre districts à l’étude sont indiqués en bleu. Les routes principales sont représentées par les lignes grises.

Figure 2 Le devis d’étude et les phases de collecte de données

**Tables**

Table 1 Statistiques descriptives des participants, par district

Table 2 Facteurs associés à la connaissance de la nouvelle politique de gratuité de la planification familiale

Table 3 Proportion des femmes utilisant la contraception qui ont dû payer pour celle0ci, avant et après l’introduction de la nouvelle politique, par région
